# B7-H3 (CD276): an actionable therapeutic target and prognostic biomarker across human malignancies

**DOI:** 10.3389/fimmu.2026.1857767

**Published:** 2026-07-17

**Authors:** Dayna Smith, Anudishi Tyagi, Venkata Lokesh Battula

**Affiliations:** 1 Research Institute of Molecular Pathology, Vienna, Austria; 2 Department of Internal Medicine, Massey Comprehensive Cancer Center, Virginia Commonwealth University, Richmond, VA, United States

**Keywords:** antibody-drug conjugates (ADCs), B7-H3, cancer immunotherapy, CAR-T cells and bispecific antibodies, immune checkpoint protein (ICP)

## Abstract

Immune checkpoint protein B7-H3 (CD276) has garnered increasing attention as a cancer immunotherapy target. Despite the absence of a definitively identified receptor, B7-H3 is implicated in multiple mechanisms of tumor progression. As a cancer biomarker, it is overexpressed in numerous cancer types and is often associated with poor prognosis. B7-H3 is currently the target of numerous immunotherapeutic strategies, and clinical trials are underway in various cancer types. This review provides an updated summary of the current body of knowledge regarding immune checkpoint protein B7-H3 and cancer therapies targeting it, highlighting its demonstrated promise as a cancer immunotherapy target, the need for further investigation into the regulation and mechanisms of B7-H3 and its associated pathways, and therapeutic progress.

## Introduction

Immunotherapy, either alone or in combination with conventional therapies like radiation or chemotherapy, represents a transformative approach in cancer treatment. Among immunotherapeutic strategies, the targeting of immune checkpoint proteins (ICPs) has demonstrated remarkable clinical success. Notable inhibitory checkpoints, including programmed cell death protein 1 (PD-1) and cytotoxic T-lymphocyte-associated protein 4 (CTLA-4), act as key regulators of immune activation but are frequently exploited by tumor cells to evade immune surveillance through altered expression (1). Inhibition of immunosuppressive ICPs by immune checkpoint inhibitors (ICIs) counteracts this suppression, restoring antitumor immunity and producing durable clinical responses in various malignancies (2). Among approved immunotherapies, ICIs remain the most promising class, with several having received Food and Drug Administration (FDA) approval (3).

Despite significant regulatory success, ICIs still face significant challenges, including resistance, limited efficacy, lack of reliable predictors of ICI response, non-durable benefits, and toxicity (4–6). Therefore, ongoing efforts to improving response rates and identifying new therapeutic targets have highlighted B7-H3, a member of the B7 family, as a promising candidate for immune checkpoint inhibition.

### B7 family

The B7 family comprises structurally related immunoregulatory proteins that provide costimulatory or coinhibitory signals to immune cells, serving as “second signals” in the two-signal model for naïve lymphocytes (7–9). Following the initial major histocompatibility complex T-cell receptor (MHC-TCR) signal between antigen presenting cell (APCs) and T-cells, these “second signal” immunoregulatory proteins regulate stimulation, inhibition, or tuning of lymphocyte response by engaging their respective receptor(s), modulating lymphocyte activation to determine the ultimate immune response (9). Depending on context, B7 family members can either enhance or suppress immune responses, maintaining immune homeostasis (8, 9). The B7 family is a critical secondary signaling mechanism essential for maintaining the balance between immune efficacy and suppression of autoimmunity (10). Despite low sequence identity, B7 family members share a conserved domain structure (11).

B7-H3 shares 20-27% amino acid identity with other B7 family members, along with the highly diverse cytoplasmic tail domain, immunoglobulin constant and variable domain (IgC- and IgV)-like domains characteristic of a B7 family member, suggesting B7-H3’s role in regulating T and natural killer (NK)-cell responses after the initial priming stage (12–14).

### Structure and isoforms of mammalian B7-H3

**Figure 1:**
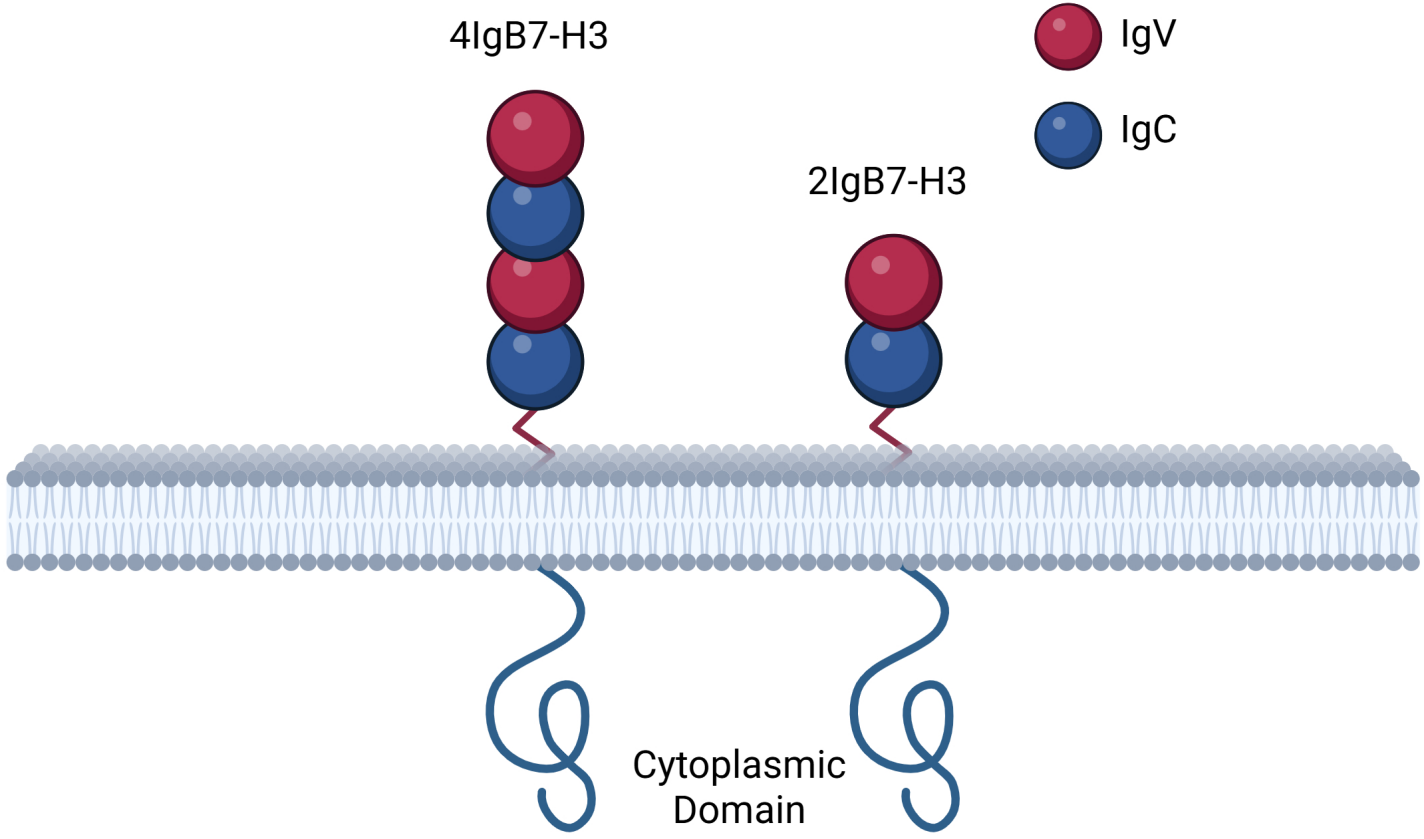
B7-H3 structure and isoforms.

B7-H3 was originally identified as a 316-amino acid type I transmembrane protein with single extracellular IgV and IgC domains, a transmembrane region, and a 45-amino acid cytoplasmic tail a highly diverse cytoplasmic domain. In humans, B7-H3 exists in two isoforms: 2IgB7-H3 and 4IgB7-H3, the latter arising from domain duplication and representing the predominant form in most normal tissues and tumor cell lines, except the brain and placenta (12, 15, 16). However, recent evidence suggests that 2IgB7-H3 may also be differentially expressed and functionally relevant in certain tumor types, highlighting the potential biological significance of both isoforms (17, 18). The duplicated IgC-IgV domain of 4IgB7-H3 may allow 4IgB7-H3 to ligate two receptors simultaneously (15). Although human and murine B7-H3 share substantial sequence homology, murine B7-H3 is expressed only as the 2Ig isoform and is encoded on chromosome 9a, whereas human B7-H3 is located on chromosome 15 (**Figure 1**) (15, 19).

In addition to its transmembrane form, B7-H3 also exists as a soluble isoform (sB7-H3), which has been detected in the serum of healthy individuals. sB7-H3 is released by both immune and tumor cells, including dendritic cells, monocytes, activated T cells, and carcinoma cells, and remains functionally active, retaining the ability to engage its yet unidentified receptor on T cells (20). Elevated sB7-H3 levels have been reported in several malignancies, including gastric adenocarcinoma, hepatocellular carcinoma, osteosarcoma, and non-muscle-invasive bladder cancer (21–24). Increased sB7-H3 expression is associated with adverse clinicopathological features, such as advanced tumor stage, invasion, metastasis, and poorer clinical outcomes (21, 23, 24).Beyond its prognostic value, sB7-H3 may also complicate therapeutic development by acting as a decoy target, potentially reducing the efficacy of therapies directed against membrane-bound B7-H3 and increasing off-target effects (25). These findings highlight the importance of disease-specific characterization of B7-H3 isoforms and the development of isoform-selective therapeutic strategies.

At the structural level, the FG loop within the IgV domain (residues 135–138) is critical for B7-H3 function. Mutational studies have shown that alteration of this region results in a near-complete loss of inhibitory activity, highlighting its importance in receptor interaction and downstream signaling (11). B7-H3 has also been shown to form dimers, a property that may influence its functional activity. Disruption of the FG loop impairs dimerization, suggesting a structural link between receptor engagement and protein assembly. Evidence from human cell models further indicates that dimerization of 4IgB7-H3 may contribute to tumorigenic signaling, although the precise mechanisms remain to be elucidated (26).

### Unknown receptor

**Figure 2:**
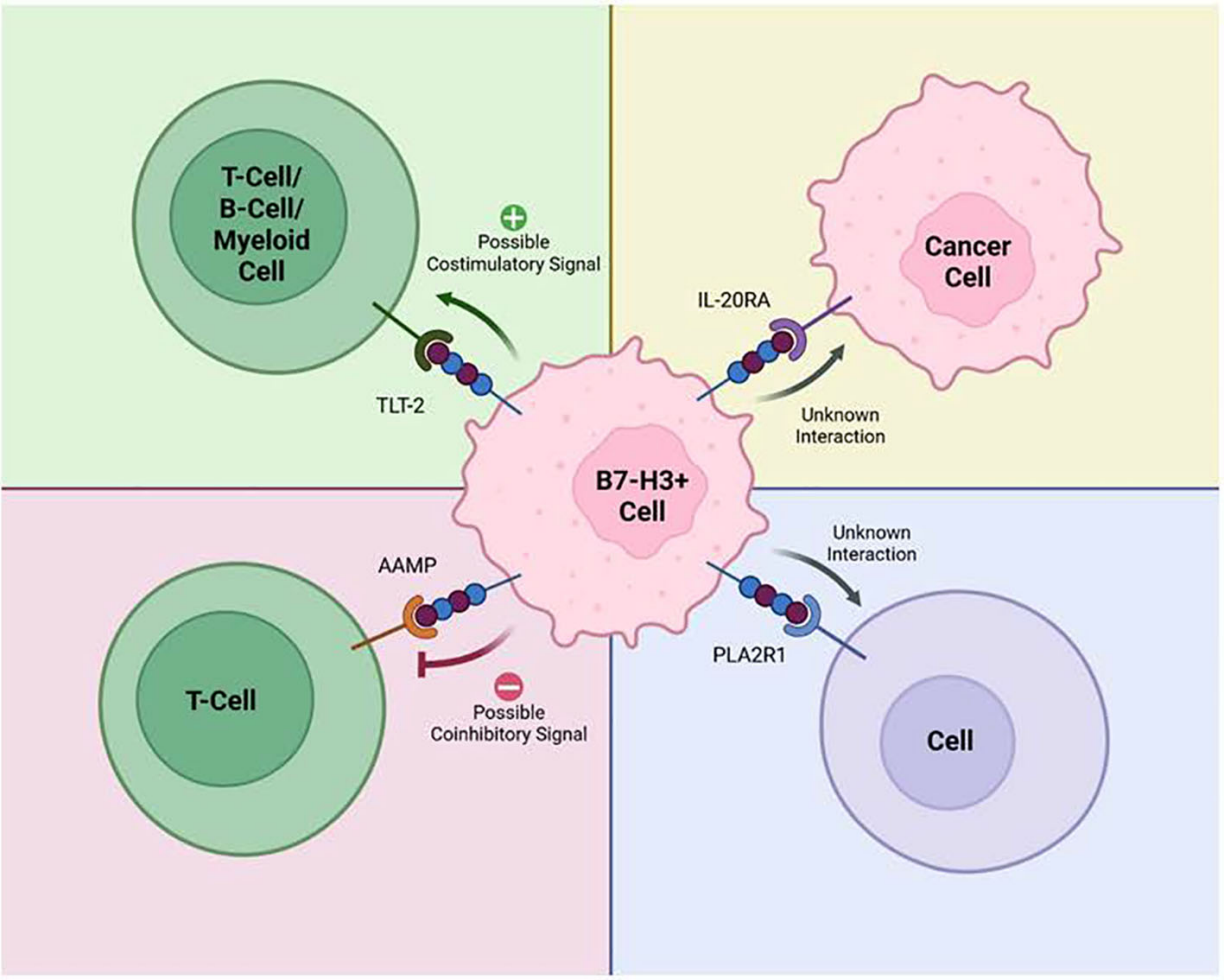
Summary of candidate B7-H3 receptors.

- Despite extensive investigation, a definitive receptor for B7-H3 has not yet been identified. Early studies proposed the existence of a counter-receptor that is transiently expressed on activated T-cells and distinct from,B7 family molecule receptors (12). Several candidates binding partners have since been suggested, although most remain controversial or incompletely validated (**Figure 2**). TREM-like Transcript-2 (TLT-2) was initially proposed as a functional receptor for T-cell based on its expression on immune cells and its reported role in T-cell activation (27, 28); however, subsequent studies failed to confirm binding, and functional data instead support an inhibitory role of B7-H3, suggesting against TLT-2 as a true receptor (11, 29, 30).
- More recently, interleukin-20 receptor subunit A (IL-20RA) was identified through extracellular interactome screening as a potential binding partner for B7-H3 (31). Although, IL-20RA has been involved promoting cancer stemness, immune evasion and an immunosuppressive microenvironment in different cancers (32–34), its role in B7-H3 signaling remains to be fully validated.
- Similarly, Phospholipase A2 receptor 1 (PLA2R1) PLA2R1 was proposed as a binding partner through vesicle-based screening platforms; however, its role in B7-H3 signaling remains speculative and requires further functional confirmation (35–37).
- Angio-associated migratory cell protein (AAMP) has also been identified as a potential binding partner for B7-H3 using screening assays where its knockdown partially reduced B7-H3-mediated suppression on T- cell proliferation, though it is unlikely to represent the sole receptor (38).

Overall, the absence of a validated receptor remains a major barrier to mechanistic understanding and therapeutic optimization. B7-H3 has been reported to possess costimulatory and coinhibitory properties, and multiple binding partners may explain its apparently opposing roles (39).

### Expression of B7-H3 and its interactions with immune cells

#### Expression in cell types and normal tissues

##### Transcriptional level expression

B7-H3 mRNA is highly expressed across human tissues including both non-lymphoid and lymphoid organs tissue. Despite this widespread transcriptional presence, expression levels vary by tissue type, with relatively lower levels observed in the brain, skeletal muscle, kidney, and lung (12). The 4IgB7-H3 isoform represents the predominant transcript in most human tissues as demonstrated by PCR analyses (15). At the transcriptional level B7-H3 expression can be upregulated by mTORC1 signaling through phosphorylation of the YY2 transcription factor via P70 S6 kinase, enhancing its stability and promoter binding. This suggests that mTORC1 hyperactivity may contribute to B7-H3 overexpression in cancer (40).

##### Posttranscriptional regulation

Although the mechanisms of the alternative splicing of B7-H3 are still unknown, the splicing factor SRSF3 has been implicated in the splicing of B7-H3 mRNA in colorectal cancer cells (41). Modes of posttranscriptional regulation that have been elucidated in cancer models include adenosine methylation at the N6 position (m6A) and microRNA (miRNA) mediated regulation (42).

##### Protein level expression

In contrast to its widespread mRNA expression, B7-H3 protein is minimally expressed in most normal tissues (43)(Flem-Karlsen et al., 2018) but is highly upregulated across a broad range of solid tumors. Immunohistochemical staining using the anti-B7-H3 mAb 8H9 have consistently revealed abundant B7-H3 tumor-associated expression with limited detection in most normal tissues supporting its tumor-selective profile (44, 45). This discordance between B7-H3 mRNA expression and B7-H3 protein suggests the involvement of post-transcriptional regulatory mechanisms. In normal tissues, its translation is tightly controlled by microRNAs, particularly miR-29 family, resulting in its limited protein expression despite having abundant mRNA transcripts (18, 46, 47). In contrast, dysregulation of these regulatory pathways in cancer, together with enhanced protein stability and tumor-associated signaling, may contribute to the overexpression of B7-H3 protein (48). This suggests the critical role of post-transcriptional regulation in controlling B7-H3 expression and potential therapeutic opportunities for targeting these regulatory mechanisms.

At the cellular level, B7-H3 localizes to multiple compartments, including exosomes and other extracellular vesicles (43). Expression is inducible on the surface of dendritic cells, monocytes, and T cells with particularly high levels of monocyte-derived immature and mature dendritic cells (12, 49).

##### Post-translational modifications

B7-H3 protein undergoes post-translational modification that influences protein stability, localization, and immunomodulatory function. B7-H3 is a highly glycosylated protein, and N-glycosylation has been shown to play a critical role in its activity. In triple-negative breast cancer (TNBC), N-glycosylation appears to contribute to B7-H3 protein stability and sustained cell-surface expression and is required for its immunosuppressive function. Core fucosylation mediated by fucosyltransferase FUT8, is particularly important in this context, with aberrant FUT8-driven glycosylation associated with enhanced immunosuppression and poor prognosis (50). Similarly, aberrant glycosylation patterns have been observed in oral cancer cells where B7-H3 N-glycans are more diverse and highly fucosylated compared with normal epithelial cells. These modifications might support carbohydrate-lectin receptor interactions with dendritic cells thereby potentially influencing immune responses (51).

##### Function and interaction with immune cells

**Figure 3:**
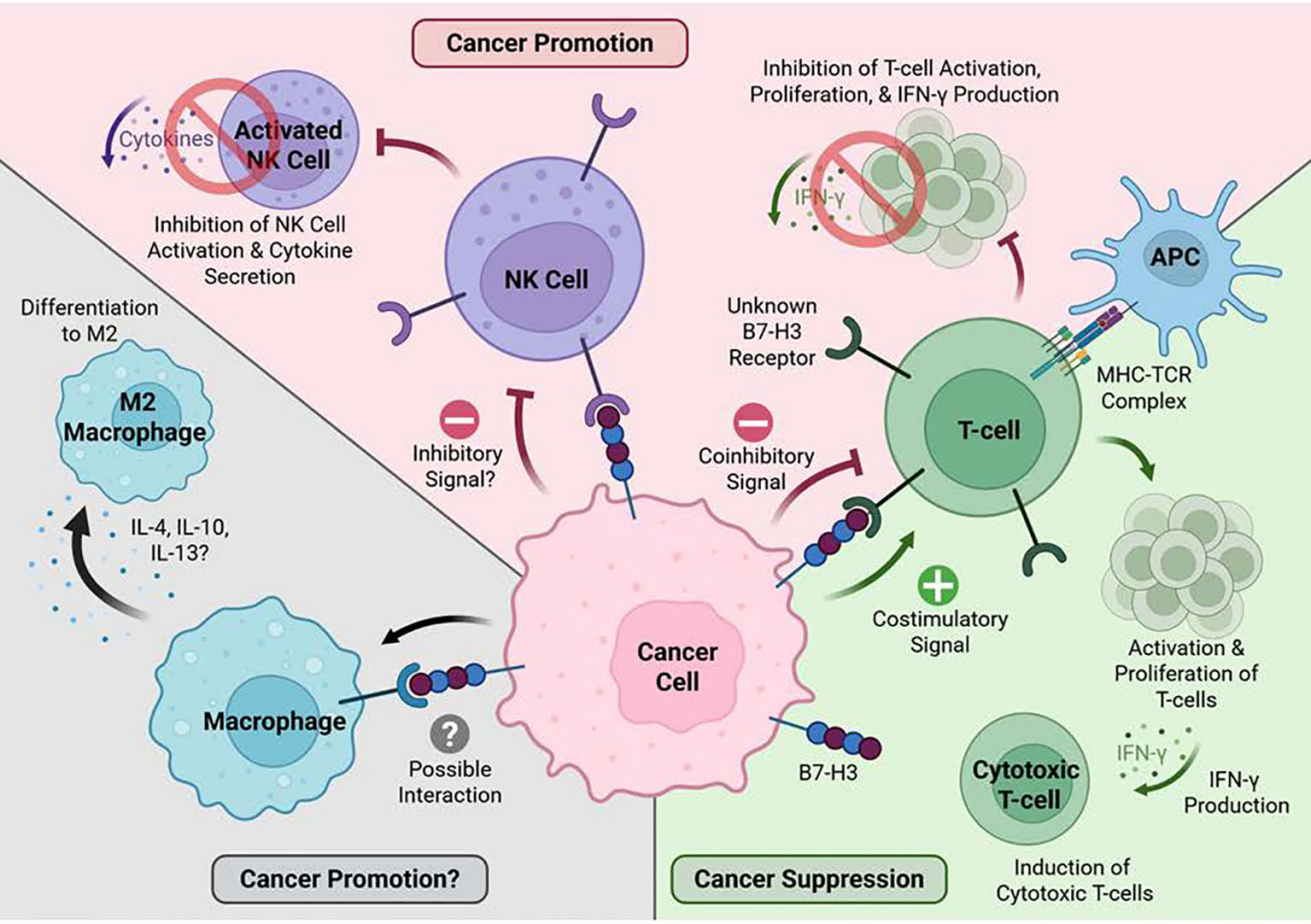
Immune interactions of B7-H3.

As a B7 family member, B7-H3 functions as an ICP, that regulates immune responses. Although initially described as a costimulatory molecule, accumulating evidence indicates that B7-H3 primarily exerts coinhibitory effects in human cancers, contributing to immune evasion (**Figure 3**).

##### Role of B7-H3 in T-cell co-stimulation

B7-H3 was initially identified as a costimulatory molecule that enhances T-cell activation. Early studies demonstrated that B7-H3 promotes the proliferation of both CD4+ and CD8+ T cells, enhances the induction of cytotoxic T cells, and increases interferon-γ (IFN-γ) production in the presence of T-cell receptor signaling (12). *In vivo*, B7-H3 has also been reported to enhance CD8+ cytotoxic T lymphocyte-mediated tumor immunity (52). This costimulatory activity has been attributed, in part, to its proposed interaction with triggering receptor expressed on myeloid cells-like TLT-2, which has been reported to enhance T-cell effector functions (29).

##### Role of B7-H3 as a coinhibitory molecule

Substantial evidence contradicts the initial characterization of B7-H3 as a costimulatory molecule and instead supports a predominantly coinhibitory role. B7-H3 inhibits T-cell activation and proliferation, as demonstrated by its suppression of both CD4+ and CD8+ T-cell responses in a dose-dependent manner and its role in downregulating T helper type 1 (Th1) immunity (30, 53). Structural studies further highlight the importance of the IgV domain FG loop in mediating this inhibitory function, with disruption of this region resulting in loss of T-cell inhibition (11). Consistent with these findings, B7-H3 inhibition enhances expansion and cytolytic activity of tumor antigen–specific CD8+ T cells, indicating that tumors exploit B7-H3-mediated signaling to evade immune surveillance (54). B7-H3 also suppresses NK cell function. B7-H3 has been identified as a neuroblastoma-associated molecule that downregulates NK-cell-mediated cytotoxicity by interacting with an unknown receptor on NK cells (49). Inhibition of B7-H3 signaling enhances NK cell activity and promotes tumor cell apoptosis across multiple cancer contexts, including neuroblastoma and acute myeloid leukemia (AML) (13, 54). These findings suggest that B7-H3 broadly suppresses cytotoxic lymphocyte responses within the tumor microenvironment.

The apparent dual costimulatory and coinhibitory functions of B7-H3 may reflect context-dependent signaling mechanisms. Proposed explanations include engagement with multiple binding partners, differential expression of alternatively spliced isoforms, and variation in post-translational modifications such as glycosylation (39, 55).

##### Other immune interactions

B7-H3 also modulates additional immune cell populations beyond T cells and NK cells, particularly macrophages and dendritic cells. B7-H3 signaling has been shown to promote polarization towards the M2 macrophage phenotype, which is associated with the immunosuppression, tumor progression, and angiogenesis (56, 57). Consistent with this, two expression markers of immunosuppressive/tumorigenic M2 macrophages positively correlated with B7-H3 expression AML (58).

Mechanistically, B7-H3 may contribute to macrophage-mediated immunosuppression through the CCL2–CCR2 axis in high-grade serous ovarian cancer, B7-H3 expression enhanced M2 macrophage migration and differentiation, and reduced IFN-γ+ CD8+ T cells collectively contributing to poorer clinical outcomes. Disruption of this axis partially abrogates the effects of B7-H3 expression in tin shaping the tumor immune microenvironment (59).

B7-H3 may also play a role in dendritic cell function, although the relationship is not well defined. Dendritic cells lacking B7-H3 exhibit enhanced capacity to activate NK and CD8^+^ T-cells (54). Additionally, glycosylated soluble B7-H3 has been shown to interact with lectin receptors on dendritic cells, suggesting a potential mechanism by which B7-H3 modulates immune responses through carbohydrate-mediated interactions (51).

#### The expression and role of B7-H3 in cancer

##### Non-immune function of B7-H3 in cancer promotion

**Figure 4:**
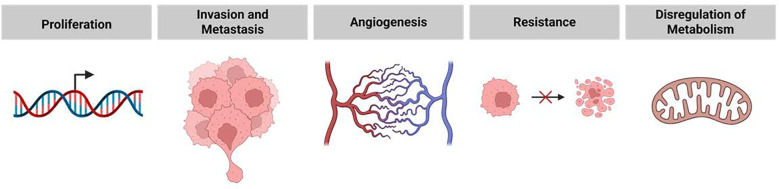
B7-H3 in cancer promotion.

Accumulating evidence indicates that tumors exploit the immunoregulatory functions of B7-H3 to evade immune surveillance, while its immune-independent roles further contribute to cancer progression. In addition to suppressing antitumor immunity, B7-H3 has been implicated in several key hallmarks of cancer, including sustained proliferative signaling, metabolic reprogramming, resistance to cell death, angiogenesis, and enhanced invasion and metastasis (60). Given its widespread overexpression across diverse malignancies and its complex regulatory mechanisms, B7-H3 may also contribute to emerging hallmarks such as nonmutational epigenetic reprogramming. Together, these findings position B7-H3 as a multifunctional driver of tumor progression that extends beyond immune modulation (**Figure 4**).

##### Proliferation

B7-H3 promotes tumor cell proliferation through regulation of cell cycle progression. Overexpression of 4Ig B7-H3 enhances proliferation and drives G1-S phase transition, accompanied by upregulation of key cell regulators (e.g., Cyclin D1, c-Myc, Pim1), Conversely, B7-H3 knockdown suppresses proliferation and induces cell cycle arrest, supporting its role in sustaining proliferative signaling (61).

##### Metabolism

B7-H3 contributes to cancer-associated metabolic reprogramming by promoting glycolysis. B7-H3 expressing cells exhibit increased glucose uptake and lactate productions when compared with vector controls, consistent with the Warburg effect (55). In contrast B7-H3 blockade shifts cellular metabolism towards oxidative phosphorylation, as evidenced by decreased basal extracellular acidification rate (ECAR), increased basal oxygen consumption rate (OCR), and resulted in lower levels of reactive oxygen species (ROS) (62). These findings suggest that B7-H3 regulates metabolic plasticity through ROS-dependent mechanisms (62).

##### Resistance

B7-H3 contributes to resistance against multiple chemotherapeutic agents by suppressing apoptosis. Elevated B7-H3 expression has been associated with reduced sensitivity to paclitaxel (PTX), cisplatin (CIS), and melphalan-induced across breast cancer, ovarian cancer, and myeloma models (61, 63, 64). Notably, inhibition of B7-H3 enhances the efficacy of these agents *in vitro* and *in vivo*, suggesting that targeting B7-H3 may overcome treatment resistance and improve therapeutic outcomes.

##### Angiogenesis, invasion and signaling pathways

B7-H3 contributes to tumor progression by promoting angiogenesis, invasion, and activation of oncogenic signaling pathways. In colorectal cancer (CRC), B7-H3 expression positively correlated with vascular endothelial growth factor A (VEGFA) and is associated with enhanced angiogenic activity (65). Mechanistically, tumor-derived exosomal B7-H3 can be internalized by endothelial cells, activating the AKT/mTOR/VEGFA signaling axis and promoting endothelial migration, invasion, and tube formation, with consistent pro-angiogenic effects observed *in vivo* (66). Additional evidence from non-cancer models further supports the role for B7-H3 in enhancing VEGF secretion (67). Across multiple, cancer types, B7-H3-high tumors are enriched for hallmark angiogenesis signatures (68).

B7-H3 also facilitates tumor cell invasion and metastasis. In hepatocellular carcinoma, elevated B7-H3 expression correlates with advanced disease features, including vascular invasion and metastasis, while its silencing suppresses invasive capacity *in vitro* (69). Consistently, transcriptomic analysis across a range of cancer types demonstrates enrichment of epithelial-mesenchymal transition (EMT) gene signatures in B7-H3 high tumors (68).

**Figure 5:**
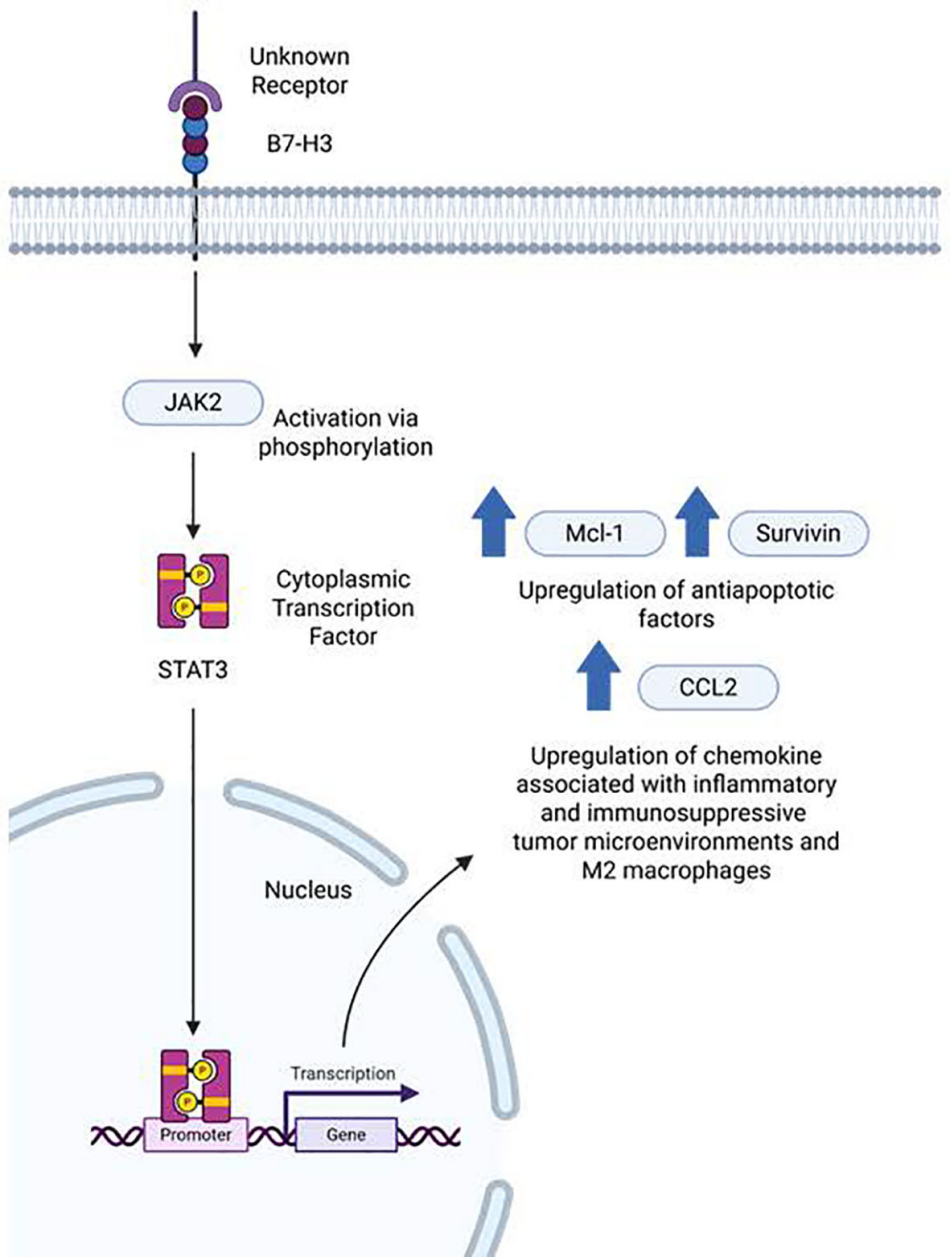
Jak2/Stat3 signaling pathway.

Mechanistically, the non-immune functions of B7-H3 are mediated through several oncogenic signaling pathways, including JAK2/STAT3, PI3K/AKT/mTOR, and NF-κB (**Figure 5**). Activation of the JAK2/STAT3 pathway promotes tumor cell survival and proliferation through upregulation of anti-apoptotic proteins such as survivin and Mcl-1, while also contributing to an immunosuppressive tumor microenvironment via CCL2 signaling (59, 61). B7-H3-mediated activation of the PI3K/AKT/mTOR axis supports cell growth, metabolic reprogramming, angiogenesis, and therapeutic resistance (66, 70). In parallel, B7-H3 stimulates NF-κB signaling, resulting in increased expression of pro-survival and pro-angiogenic factors, including IL-8, Bcl-2, cyclin D1, COX-2, and VEGFA (64, 65). Together, these pathways provide the molecular framework through which B7-H3 promotes proliferation, metabolic adaptation, treatment resistance, angiogenesis, invasion, and metastasis.

##### Expression in various cancer types

###### Regulation of expression in cancer

**Figure 6:**
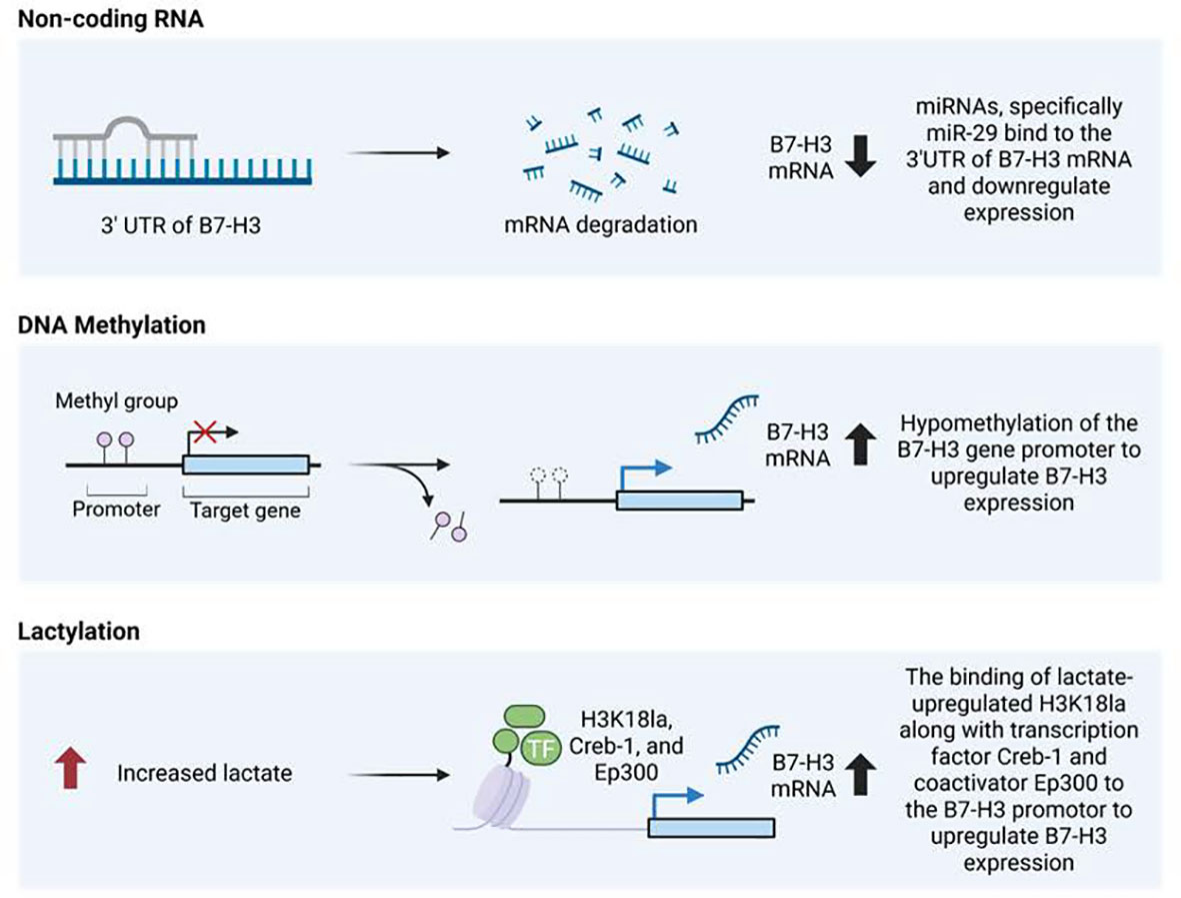
Possible regulators of expression.

B7-H3 expression is regulated by multiple epigenetic and post-transcriptional mechanisms, including miRNAs, DNA methylation, and histone modifications (**Figure 6**). Several miRNAs, non-coding RNA molecules that bind to 3’UTRs of mRNAs to regulate gene expression, have been identified as negative regulators of B7-H3 expression, including miR-143, miR-187, miR-29, and miR-124 (71). Among these miR-29 has been shown to directly target the 3′UTR of B7-H3 mRNA, leading to its degradation and reduced protein expression, with downstream effects on immune activation such as enhanced NK cell activity (44, 47).

DNA methylation has also been implicated in the regulation of B7-H3, although its role appears to be context-dependent. Promoter hypomethylation has been associated with increased B7-H3 expression in certain cancers, including glioma (72), potentially related to the grade and type of glioma (73). However, other studies, such as in AML, suggest that B7-H3 expression may be largely independent of promoter methylation status (58). These findings indicate that methylation-dependent regulation of B7-H3 may vary across tumor types. While methylation as a potential regulator of B7-H3 expression in cancer needs further investigation, a study of ankylosing spondylitis, which may also be characterized by elevated B7-H3 mRNA expression, also suggested hypomethylation of the B7-H3 promoter may increase B7-H3 expression. Although a relatively weak correlation between mRNA expression (protein expression was not measured) and methylation of the B7-H3 gene was observed (74). It may be that methylation is a notable regulator of B7-H3 only in certain cancer types, excluding AML. Further investigation of the role of methylation in B7-H3 expression in cancer models is warranted.

In addition to DNA-level regulation, post-transcriptional RNA modifications contribute to B7-H3 expression. Adenosine methylation at the N6 position (m6A), a common eukaryotic posttranscriptional modification, has been shown to stabilize B7-H3 mRNA through the m6A reader YTHDF1, promoting tumor growth and metastasis in colorectal cancer (42).

Histone modifications further regulate B7-H3 expression. Histone lactylation, a modification linked to metabolic reprogramming in cancer, has been shown to enhance B7-H3 transcription through recruitment of transcriptional regulators such as CREB-1 and EP300 to its promoter (75, 76).

Collectively, these regulatory mechanisms underscore the complexity of B7-H3 expression control and suggest that epigenetic and post-transcriptional pathways may represent viable therapeutic targets. Targeting these regulatory axes (e.g., DNA methylation, histone modifications, non-coding RNA expression) could provide additional strategies to modulate B7-H3 expression and enhance the efficacy of B7-H3-directed therapies (77, 78).

##### Differential expression of B7-H3 isoforms

Differential expression of the 2IgB7-H3 and 4IgB7-H3 isoforms have been observed across cancer types, and may carry diagnostic and prognostic significance. In general, 4IgB7-H3 is the predominant isoform and is widely expressed across tumor cell lines, whereas 2IgB7-H3 exhibits more restricted expression (79). Notably, 2IgB7-H3 appears to have greater tumor specificity in certain contexts. In glioma, 2IgB7-H3 is expressed in tumor tissue but is largely absent in normal brain, whereas 4IgB7-H3 is detectable in both, suggesting that the 2Ig isoform may offer improved diagnostic specificity (79). Similarly, in AML, 2IgB7-H3 mRNA expression is elevated and distinguishes patients from controls, while 4IgB7-H3 expression shows no significant difference. Higher expression of 2IgB7-H3 is also associated with poorer overall survival, further supporting its potential prognostic value (80). Collectively, these findings suggest that while 4IgB7-H3 is the broadly expressed and predominant isoform in many human tumor cell lines (12, 15, 16), 2IgB7-H3 may be a more useful as a disease-specific biomarker in certain malignancies. Further characterization of isoform-specific expression patterns may enhance diagnostic precision and inform targeted therapeutic strategies.

##### Upregulated or high expression is observed in various cancer types

One of the most compelling contributors to B7-H3’s promise as a therapeutic target is its widespread overexpression across diverse malignancies, together with minimal protein expression in normal tissues. Elevated B7-H3 levels have been reported in numerous solid tumors including glioblastoma, prostatic adenocarcinoma, and head and neck squamous cell carcinoma (HNSCC), whereas, its expression is low in normal tissues (47, 72, 81). Notably, in prostate cancer, B7-H3 is also highly expressed, with a median of 80% of tumor cells showing positive in large patients cohorts (82). This tumor-selective expression profile supports its potential utility as both a biomarker and a target for immunotherapy, including in contexts where B7-H3 may may contribute to immune escape and resistance to conventional ICIs (81). Despite this tumor-selective expression pattern, its expression is frequently heterogeneous both between patients and within individual tumors. Such inter- and intra-tumoral variability represents a key challenge for targeted therapies, as heterogeneous B7-H3 expression may limit uniform therapeutic efficacy and contribute to variable clinical responses. Therefore, patient stratification based on B7-H3 expression may be important to optimize therapeutic outcomes. In addition, various combinations strategies should be used to address intra-tumoral heterogeneity e.g. combination with anti-PD1/PD-L1 inhibitors.

##### Association of expression with poor prognosis

High B7-H3 expression is consistently associated with adverse clinical outcomes across a broad range of cancer types. Large scale analyses encompassing multiple malignancies demonstrate that elevated B7-H3 expression correlates with reduced overall survival in cancers such as colorectal, gastric, bladder, ovarian, lung, and hepatocellular carcinoma, among others (68). These findings are supported by tumor-specific studies where elevated B7-H3 expression is linked to more aggressive disease features, including metastasis, recurrence, and decreased survival in cancers such as neuroblastoma, glioma, prostate cancer, hepatocellular carcinoma, and multiple myeloma (47, 69, 72, 83). Collectively, these data establish B7-H3 as a broadly overexpressed, prognostically unfavorable biomarker that reflects tumor aggressiveness and immune evasion, further supporting its role as a clinically relevant therapeutic target.

### Therapeutic opportunities to target B7-H3 in cancer

#### Blocking and ADCC-Inducing mAbs

**Figure 7:**
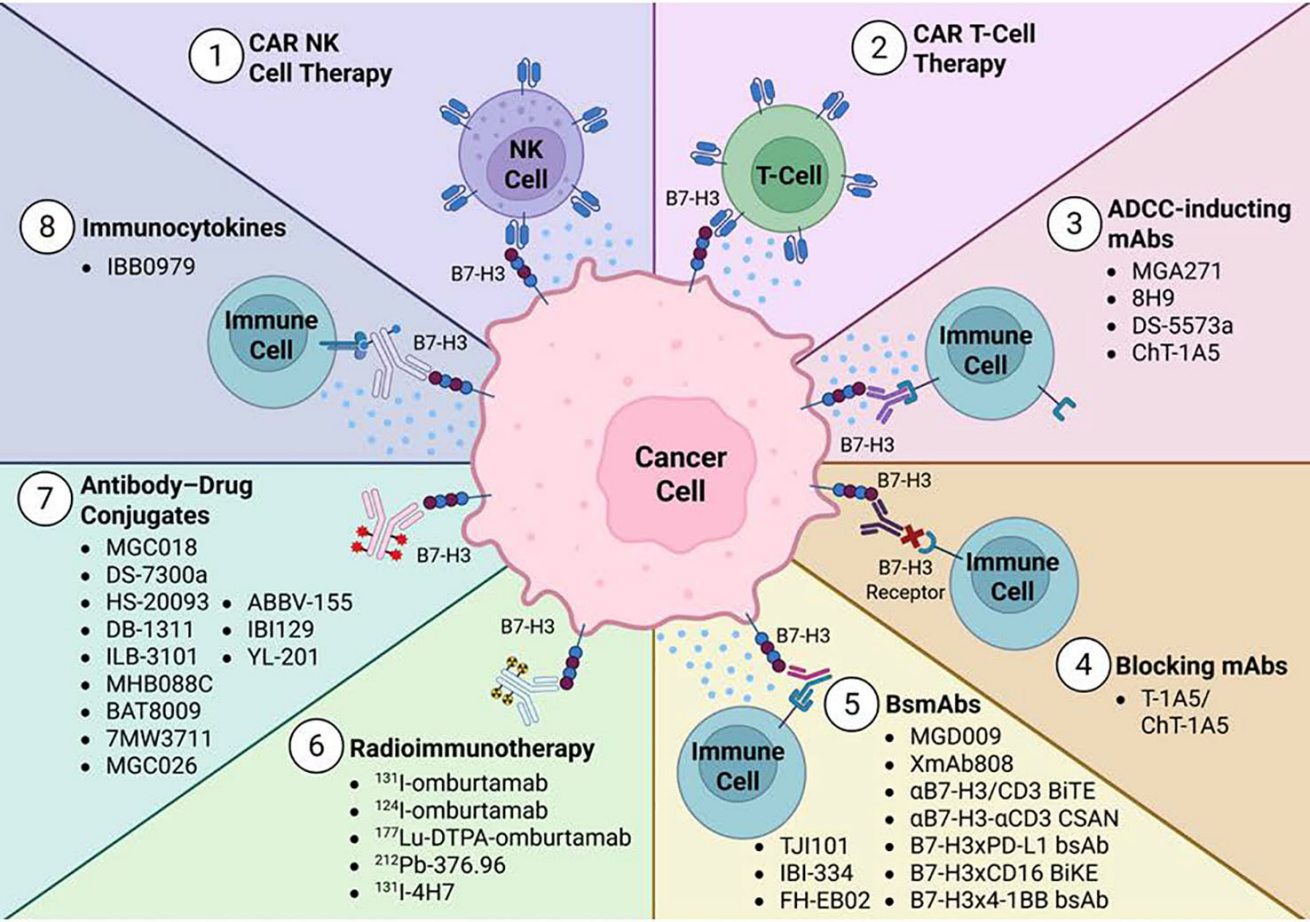
Notable antibody-based therapies against B7-H3.

Monoclonal antibodies targeting B7-H3 represent a key therapeutic strategy, functioning through blockade of immunosuppressive signaling and antibody-dependent cellular cytotoxicity (ADCC). Although the B7-H3 receptor remains undefined, these antibodies are designed to directly target B7-H3 expressing tumor cells and enhance immune-mediated killing (**Figure 7**).

Preclinical studies have demonstrated that antibodies such as T-1A5 which binds the FG loop of B7-H3, can restore anti-tumor immunity by enhancing NK cell mediated cytotoxicity, while also inducing ADCC in B7-H3-positive malignancies (13, 78, 84).

Enoblituzumab (MGA271), a humanized Fc-engineered anti-B7-H3 mAb, represents the most clinically advanced agent in this class. Designed to enhance binding to activating Fc receptors CD16A and reduce binding to inhibitory receptor CD32B, enoblituzumab has demonstrated ADCC activity across multiple tumor models and favorable safety profiles (85). Clinical studies including combination approaches with PD-1 inhibitors, (e.g., NCT01391143, NCT02982941, NCT02923180, NCT02475213, NCT02381314, NCT04129320, NCT04634825, NCT04630769, NCT06014255) have shown modest response rates with evidence of immune activation, although efficacy appears variable depending on prior checkpoint inhibitor exposure (86). In a phase I combination study of MGA27I with anti-PD-1 mAbs (NCT02475213), MGA271 in combination with pembrolizumab resulted in treatment-related adverse events in 116/133 patients (87.2%), of which 28.6% were grade ≥3. Objective responses occurred in 6 of 18 (33.3%) patients with anti-PD-1/PD-L1-naïve HNSCC and in 5 of 14 (35.7%) patients with anti-PD-1/PD-L1-naïve non-small cell lung cancer (NSCLC). Patients with prior anti-PD-1/PD-L1 treatment demonstrated poorer overall survival, with reduced ORRs among those with NSCLC and no responses among those with HNSCC. Limited responses were observed among patients with urothelial cancer and melanoma (87). Another combination study, a phase II trial of MGA271 in combination with either anti-PD-1 mAb (MGA012) or a PD-1 × LAG-3 BsAb DART (MGD013), was terminated following an internal review of safety data (NCT04634825). Results obtained prior to the study’s closure are available on Clinicaltrials.gov and indicate that of the MGA271 with MGA012 cohort of 48 patients, no patients demonstrated a CR, 6.3% (3/48) demonstrated a PR, and 41.7% (20/48) demonstrated SD for at least 3 months. AEs were observed in 83.3% (40/48) of patients in the cohort, while 29.17% (14/48) were affected by serious adverse events. In the MGA271 with MGD013 cohort of 14 patients, no patients demonstrated a CR, 14.3% (2/14) demonstrated a PR, and 35.7% (5/14) demonstrated SD for at least 3 months. AEs were observed in 85.7% (12/14) of patients in the cohort, while 42.86% (6/14) were affected by/at risk for serious adverse events. A study of MGA271 in pediatric patients with relapsed or refractory solid tumors (NCT02982941) and a study of MGA271 in combination with ipilimumab, an anti-CTLA-4 mAb, in patients with melanoma, NSCLC, and other cancers (NCT02381314) have both been completed, but results are yet to be released.

Other ADCC-enhancing antibodies, such as the afucosylated antibody DS-5573a, have demonstrated both ADCC and antibody-dependent cellular phagocytosis (ADCP) activity in preclinical models, although clinical development has been limited (88).

#### Radioimmunotherapy

Radioimmunotherapy represents a complementary strategy for targeting B7-H3 by delivering cytotoxic radiation directly to tumor cells. Omburtamab (8H9), labeled with radioisotopes such as iodine-131 (^131^I-omburtamab;131 I-8H9) has shown encouraging clinical activity, particularly in central nervous system malignancies (NCT00089245) (**Figure 7**). However, the favorable disease control rates and prolonged survival in subsets of patients, have been accompanied by hematologic toxicities, including myelosuppression, thereby highlighting a key limitation (89, 90). Additional radiolabeled variants, including lutetium-177 conjugates, have been evaluated in early-phase studies with acceptable safety profiles, while several preclinical agents continue to demonstrate antitumor activity in solid tumor models (91–94). Furthermore, Furthermore, although iodine-124 is primarily a positron emission tomography (PET) imaging radionuclide, 124I-omburtamab has been investigated as a theranostic agent that combines tumor imaging, dosimetry, and localized radiation delivery in diffuse intrinsic pontine glioma (NCT01502917) (95, 96). In addition to radiotherapy, PET based imaging of B7-H3 is an emerging approach for non-invasive tumor assessment. Recent preclinical studies have developed novel radiotracers based on affibody and nanobody platforms, which offer high affinity, rapid tumor targeting, and favorable imaging contrast (97–99). These agents may enable sensitive detection of B7-H3 expressions and support patient stratification and treatment monitoring for B7-H3-targeted therapies. Although still in early preclinical stages, these imaging platforms represent a promising direction for the clinical translation of precision immuno-oncology.

#### Antibody-drug conjugates

Antibody-drug conjugates (ADCs) represent one of the most advanced and promising therapies targeting B7-H3 in various cancer types. These agents combine the tumor specificity of monoclonal antibodies with the cytotoxic potency of chemotherapeutic payloads, enabling targeted delivery of drugs to B7-H3-expressing tumor cells. Most B7-H3-directed utilize DNA-damaging payloads, particularly topoisomerase inhibitors, which induce cell cycle arrest and apoptosis.

Several B7-H3-targeting ADCs have demonstrated robust antitumor activity in both preclinical and clinical settings. MGC018 (vobramitamab duocarmazine or vobra duo) a duocarmycin-based ADC has demonstrated antitumor activity across multiple solid tumors, and is currently under clinical evaluation, including in combination with ICIs (100–102) (**Figure 7**).

Similarly, HS-20093 and YL201 both conjugated to topoisomerase inhibitor payloads, have demonstrated encouraging response rates and disease control in early-phase clinical trials across a range of malignancies, including small cell lung cancer and nasopharyngeal carcinoma (75, 103).

ABBV-155 (Mirzotamab clezutoclax or Mirzo-C) is a B7-H3-directed ADC conjugated to a BCL-XL inhibitor payload. A phase I trial of ABBV-155 alone or in combination with taxanes in breast cancer, SCLC, and NSCLC (NCT03595059) has been completed, with final results still pending. Preliminary results suggest a tolerable safety profile, with ORRs of 0% (0/14), 11% (4/36), and 18% (5/28) in the SCLC, NSCLC, and breast cancer cohorts, respectively. Results from *in vivo* experiments indicate that ABBV-155 may also be a candidate for combination therapy with venetoclax in AML, with combination therapy in PDX mouse models inhibiting leukemia burden and prolonging survival (104).

Ifinatamab deruxtecan (I-DXd, DS-7300a) (105), is among the most clinically advanced agents in this class. Clinical studies have reported objective response rates ranging from approximately 25% to over 50% across tumor types, with particularly strong activity in small cell lung cancer (101, 106, 107). These findings have led to the granting of Breakthrough Therapy Designation by the FDA, underscoring the therapeutic potential of B7-H3-directed ADCs in heavily pretreated populations (108, 109).

Additional ADCs, including ABBV-155, DB-1311, MHB088C, and 7MW3711, have shown varying degrees of clinical or preclinical activity, with some demonstrating dual mechanisms such as ADCC or ADCP (110). While safety profiles are generally manageable, hematologic toxicities, i.e., neutropenia and thrombocytopenia, remain common across this class.

Despite these promising developments, several challenges remain. Notably, the relationship between B7-H3 expression levels and treatment response is not yet clearly defined, and resistance mechanisms to ADC-based therapies are poorly understood. Ongoing clinical trials and combination strategies will be critical to optimizing the efficacy of B7-H3-targeted ADCs and defining their role within the broader immuno-oncology landscape.

#### Bispecific agents

Bispecific antibodies represent an emerging strategy to enhance the therapeutic targeting of B7-H3 by simultaneously engaging tumor cells and immune effector pathways. These agents are designed to redirect immune cells, most commonly T cells or NK cells, toward B7-H3-expressing tumors or to provide localized costimulatory signals within the tumor microenvironment (**Figure 7**).

The most extensively studied class includes B7-H3xCD3 bispecifics, which recruit and activate T cells to induce tumor cell killing. CD3 provides the first signal of lymphocyte activation sensed by the TCR, and modulation of CD3-TCR function is a therapeutic area of interest in cancer treatment (111). Agents such as MGD009 (a DART platform molecule) have demonstrated the ability to promote T-cell infiltration, activation, and cytotoxicity in preclinical models, with enhanced activity observed in combination with PD-1 blockade (112). Additional B7-H3×CD3 constructs, including conventional bispecific T-cell engagers and novel platforms such as chemically self-assembling nanorings (CSANs), have shown potent antitumor activity across multiple tumor types, including glioblastoma and gastrointestinal cancers (19, 113, 114).

Beyond CD3-based approaches, alternative bispecific strategies aim to modulate immune costimulation or engage additional immune cell populations. B7-H3xCD28 bispecifics are designed to provide localized T cell costimulation, While B7-H3xCD16 bispecific killer cell (BiKE) constructs enhance NK cell-mediated cytotoxicity. Similarly, B7-H3x4-1BB bispecific antibody demonstrated the ability to induce a potent 4-1BB-dependent anti-tumor response in B7-H3-overexpressing tumor models without systemic toxicity and appears to inhibit tumor growth synergistically with PD-1 blockade (34, 62).

Another area of active development is bispecific antibodies targeting B7-H3 in combination with PD-L1 or epidermal growth factor receptor (EGFR). These dual-targeting approaches aim to enhance tumor specificity, i.e., to more selectively inhibit EGFR signaling in tumor cells and overcome limitations of single-agent therapies. Preclinical studies suggest that B7H3×PD-L1 and B7-H3×EGFR bispecifics exhibit improved antitumor activity and reduced off-target toxicity. compared with monotherapies, highlighting their potential for combination-based targeting strategies (115, 116). Despite promising preclinical results (117, 118), most B7-H3-targeting bispecific agents remain in early clinical development. Key challenges include optimizing safety, particularly with respect to cytokine release, and identifying patient populations most likely to benefit. Nonetheless, these approaches offer a flexible platform for integrating immune activation with tumor-specific targeting and represent a rapidly evolving area of B7-H3-directed therapy.

### CAR-T cells

B7-H3-targeting chimeric antigen receptor T cells (CAR-T cells) are being actively investigated across multiple solid tumors, reflecting the broad expression of B7-H3 and its tumor-restricted protein profile.

Early clinical studies, such as those evaluating the allogeneic CAR-T product, MT027, in recurrent high-grade glioma (ChiCTR2100047968), have demonstrated acceptable safety, and preliminary antitumor activity, with evidence of sustained CAR-T cell persistence and immune activation (119). These findings support the feasibility of targeting B7-H3 in solid tumors, although challenges related to durability of response, tumor infiltration, and safety remain. Recent evidence has shown that certain anti-B7-H3 CAR-T cells may induce on-target/off-tumor toxicity, likely due to low-level expression of B7-H3 in normal tissues. These findings highlight the importance of CAR design and antigen threshold sensitivity in ensuring safety (97).

### Additional therapies

Other therapeutic opportunities that might effectively target B7-H3 include immunocytokines, CAR-NK cells, hairpin RNAs/RNA interference, and miRNAs. *In vivo* experiments with CAR-NK cells in non-small cell lung cancer models demonstrate promising results (120). IBB0979 is a B7-H3/IL-10 immunocytokine (antibody-cytokine fusion protein) that has also reached the clinical trial phase. Notable clinical trials are summarized in **Tables 1** and **2**.

**Table 1:** Clinical trials targeting B7-H3 without outcomes data.

Trial identifier (Phase)	Drug/treatment	Treatment type	Cancer type
NCT01391143 (I) NCT02982941 (I) NCT02923180 (II) NCT06014255 (II)	MGA271 (Enoblituzumab)	ADCC-inducing mAb	Refractory cancer; relapsed or refractory solid tumors; prostate cancer; prostate cancer
NCT02475213 (I)	MGA271 (Enoblituzumab) + Pembrolizumab/MGA012	ADCC-inducing mAb with anti-PD-1 mAb	Melanoma, head and neck cancer, non-small cell lung cancer, urothelial carcinoma
NCT02381314 (I)	MGA271 (Enoblituzumab) + Ipilimumab	ADCC-inducing mAb with anti-CTLA-4 mAb	Melanoma, non-small cell lung cancer
NCT04129320 (II/III) NCT04634825 (II)	Enoblituzumab + Retifanlimab (MGA012) or Tebotelimab (MGD013)	ADCC-inducing mAb with anti-PD-1 mAb or PD-1 X LAG-3 BsAb DART	Head and neck cancer, head and neck squamous cell carcinoma; head and neck cancer, head and neck neoplasms, head and neck squamous cell carcinoma
NCT04630769 (I)	Enoblituzumab + FT516 + IL-2	ADCC-inducing mAb with NK cell therapy (FT516 - NK cells engineered with high-affinity, non-cleavable CD16) and Interleukin-2	Ovarian cancer, fallopian tube adenocarcinoma, primary peritoneal cavity cancer
NCT02192567 (I)	DS-5573a	ADCC/ADCP-inducing mAb	Advanced solid malignant tumors
NCT00089245 (I) NCT03275402 (II/III) NCT01099644 (I) NCT04743661 (II) NCT00582608 (N/A) NCT05063357 (I) NCT05064306 (Expanded access)	^131^I-omburtamab (131 I-8H9)	Radioimmunotherapy	Brain and CNS tumors, neuroblastoma, sarcoma; neuroblastoma, CNS metastases, leptomeningeal metastases; peritoneal cancer; recurrent medulloblastoma, recurrent ependymoma; CNS cancer, neuroblastoma, sarcoma; DIPG; CNS/leptomeningeal neoplasms
NCT04022213 (II)	^131^I-omburtamab (131 I-8H9) + WAP-IMRT	Radioimmunotherapy with radiation therapy	Peritoneal cancer
NCT01502917 (I)	^124^I-omburtamab	Radioimmunotherapy	Brain cancer, brain stem glioma,DIPG
NCT04167618 (I/II) NCT04315246 (I/II)	177Lu-DTPA-omburtamab	Radioimmunotherapy	Childhood medulloblastoma; leptomeningeal metastasis, adult solid tumors
NCT06242470 (I)	MGC026	ADC	Relapsed/refractory, unresectable, locally advanced, or metastatic solid tumors (121)
NCT05293496 (I)	MGC018 (vobramitamab duocarmazine) + MGD019 (lorigerlimab)	ADC with PD-1 x CLTA-4 Dual-Affinity Re-Targeting (DART) protein (BsAb)	Advanced solid tumors
NCT05551117 (II)	MGC018 (vobramitamab duocarmazine)	ADC	Prostatic cancer
NCT04145622 (I/II) NCT05280470 (II) NCT06203210 (III) NCT06330064 (II) NCT06644781(III) NCT06362252 (I/II)* NCT06780137 (I/II)*	DS-7300a (Ifinatamab Deruxtecan or I-DXd)	ADC (* - in combination with other agent(s))	Advanced solid tumors, malignant solid tumors; ES-SCLC; SCLC; recurrent metastatic solid tumors; esophageal squamous cell carcinoma (ESCC); ESCLC; SCLC
NCT06112704 (II) NCT05830123 (II) NCT05276609 (I) NCT06052423 (II) NCT06001255 (II) NCT06007729 (II) NCT06332170 (I)* NCT06699576 (I)* NCT06825624 (I)*	HS-20093	ADC (* - in combination with other agent(s))	Advanced solid tumors; osteosarcoma, sarcoma; advanced solid tumors; ES-SCLC; mCRPC; head and neck squamous cell carcinoma; advanced solid tumors; osteosarcoma, soft tissue sarcoma; metastatic colon cancer
NCT05914116 (I/II)	DB-1311 (BNT324)	ADC	Advanced solid tumors
NCT06953089 (II)	DB-1311 (BNT324) with BNT327 or DB-1305 (BNT325)	ADC with PD-L1/VEGF BsAb (BNT327) or with anti-TROP2 ADC (DB-1305)	Solid tumors
NCT06892548 (I/II)	DB-1311 (BNT324) with BNT327	ADC with PD-L1/VEGF BsAb	Advanced lung cancers
NCT06629597 (III) NCT06612151 (III) NCT06241846 (II) NCT06394414 (I) NCT07258979 (I/II)* NCT07208773 (I/II)* NCT06898957 (I)* NCT07307053 (I/II)	YL201	ADC (* - in combination with other agent(s))	Recurrent or metastatic nasopharyngeal carcinoma; relapsed SCLC; mCRPC; advanced solid tumors; recurrent or metastatic nasopharyngeal carcinoma; advanced solid tumors, SCLC, and NSCLC; ES-SCLC; rare solid tumors
NCT05652855 (I/II) NCT06951243 (II) NCT06954246 (III)	MHB088C	ADC	Advanced or metastatic solid tumors; advanced extrapulmonary neuroendocrine cancer; ES-SCLC
NCT05405621 (I)	BAT8009	ADC	Advance solid tumors
NCT06426680 (I)	ILB-3101	ADC	Advanced solid tumors (119)
NCT05991349 (I/II)	IBI129	ADC	Unresectable, locally advanced, or metastatic solid tumors
NCT06736327 (I)	SKB500	ADC	Advance solid tumors
NCT07296809 (II)	SKB500 + KL-A1267, carboplatin, and etoposide	ADC	Lung cancer
NCT02628535 (I)	MGD009	DART protein (BsAb)	Various B7-H3-expressing tumors
NCT03406949 (I)	MGD009 + MGA012	DART protein (BsAb) with anti-PD-1 mAb	Advanced solid tumors
NCT05585034 (I)	XmAb808 + pembrolizumab	B7-H3 X CD28 BsAb with anti-PD-1 mAb	Advanced solid tumors (122)
NCT05774873 (I/II)	IBI-334	B7-H3 X EGFR BsAb	Unresectable, locally advanced, or metastatic solid tumors
NCT06349408 (I)	IBI3001	BsAb ADC (IBI-334 conjugated to exatecan)	Unresectable, locally advanced, or metastatic solid tumors (116)
NCT07181473 (I)	TJ101	B7-H3 X EGFR BsAb ADC with PY-4car2 payload	advanced/metastatic solid tumors
NCT05991583 (I/II)	IBB0979	B7-H3/IL-10 immunocytokine	Advanced malignant tumors
NCT04897321(I)	B7-H3 CAR T cells	CAR T Cell	Pediatric solid tumors
NCT04077866 (I/II)	B7-H3 CAR T cells	CAR T Cell	Recurrent glioblastoma, refractory glioblastoma
NCT05515185 (I)	B7-H3 KT095 CAR T cells	CAR T Cell	Advanced solid tumors
NCT04385173 (I)	B7-H3 CAR T cells	CAR T Cell	Recurrent glioblastoma, refractory glioblastoma
NCT05474378 (I)	B7-H3 CAR T cells	CAR T Cell	Recurrent glioblastoma multiforme
NCT06500819 (I)	B7-H3 CAR T cells	CAR T Cell	Relapsed or refractory solid tumors: neuroblastoma, sarcoma osteosarcoma
NCT06646627 (I)	B7-H3 CAR T cells	CAR T Cell	Ovarian cancer
NCT05835687 (I)	B7-H3 CAR T cells	CAR T Cell	Pediatric primary CNS tumors
NCT06158139 (I) NCT06305299 (I) NCT06347068 (I)	iC9-CAR.B7-H3 T cells	CAR T Cell containing inducible caspase 9 safety switch (iC9)	Refractory pancreatic ductal adenocarcinoma; ovarian cancer; relapsed/refractory triple-negative breast cancer
NCT04670068 (I)	B7-H3 CAR T cells	CAR T Cell	Epithelial ovarian cancer
NCT05241392 (I)	B7-H3 CAR T cells	CAR T Cell	Glioblastoma
NCT05366179 (I)	B7-H3 CAR T cells	CAR T Cell	Glioblastoma multiforme
NCT05752877 (N/A)	B7-H3 UCAR-T cells (or IL-13 Rα2 UCAR-T cells)	CAR T Cell	Advanced glioma
NCT03198052 (I)	B7-H3 CAR T cells (and 12 other CAR-Ts) with CD4+ T cells engineered to express TGFβ-CAR and secret IL7/CCL19 and/or SCFVs against PD1/CTLA4/Tigit	CAR T Cell	Advanced lung cancer or other cancer
NCT05143151 (I/II)	B7-H3 CAR T cells	CAR T Cell	Advanced pancreatic carcinoma
NCT04185038 (I)	B7-H3 CAR T cells (SCRI-CARB7H3)	CAR T Cell	CNS tumors, DIPG
NCT05768880 (I)	B7-H3/EGFR806/HER2/IL13-zetakine (Quad) CAR T cells (SC-CAR4BRAIN)	Multi-antigen-targeted CAR T Cell	DIPG, DMG, adult and pediatric recurrent CNS tumors, refractory primary malignant CNS neoplasm
NCT04637503 (I/II)	GD2/PSMA/B7-H3 CAR T cells	Multi-antigen-targeted CAR T Cell	Neuroblastoma
NCT04864821 (I)	B7-H3 CAR T cell	CAR T Cell	Osteosarcoma, neuroblastoma, gastric cancer, lung cancer
NCT04483778 (I)	B7-H3 CAR T cells (SCRI-CARB7H3) tracking/suicide construct EGFRt + B7-H3/CD19 CAR T cells (SCRI-CARB7H3x19) with tracking/suicide construct HER2tG + prembrolizumab	CAR T Cell/Multi-antigen-targeted CAR T Cell with anti-PD-1 mAb	Pediatric solid tumor
NCT04842812 (I)	B7-H3 CAR-TILs (and 13 other CAR-TILs and TILs engineered to knockdown PD1 and express scFvs that target PD1 and CTLA4)	CAR-TIL	Advanced cancers (liver cancer, lung cancer, breast cancer, CRC, brain tumor, adult solid tumor)
NCT04432649 (I/II)	B7-H3 CAR T cells (4SCAR-276)	CAR T cell	Solid tumors
NCT05562024 (I) NCT05190185 (I)	TAA06	CAR T Cell	Relapsed/refractory neuroblastoma; malignant melanoma, lung cancer, CRC
NCT05731219 (I) NCT05722171 (I) NCT06372236 (I)	UTAA06	CAR T Cell	Relapsed/refractory AML; relapsed/refractory AML; relapsed/advanced malignant solid tumors
NCT06221553 (I) NCT06612645 (I)	CMD03DIPG; CMD03	B7H3-IL7Ra CAR-T cells	DIPG; pediatric cancers, solid tumor pediatric
NCT06482905 (I)	TX103	CAR T Cell	Recurrent or progressive grade IV glioma
NCT06742593 (I) NCT06912152 (I) NCT07004647 (N/A)	MT027	Off-the-shelf, allogeneic CAR-T (UCAR-T) cells targeting B7-H3	Brain (nervous system) cancers, brain and CNS tumors; advanced primary peritoneal tumors or secondary abdominal metastatic malignant solid tumors

**Table 2:** Clinical trials targeting B7-H3 with outcomes data.

Trial identifier (Phase)	Drug/treatment	Treatment type	Cancer type	Results
NCT01391143 (I)	MGA271 (Enoblituzumab)	ADCC-inducing mAb	Refractory cancer (multiple types)	MGA271 was well-tolerated. Patients experienced disease stabilization and tumor shrinkage across several tumor types (123).
NCT02923180 (II)	MGA271 (Enoblituzumab)	ADCC-inducing mAb	Prostate cancer	Treatment was well tolerated. Possible enhanced clinical outcomes. Association between treatment and net grade group decrease. TME profiling was indicative of an inflammatory antitumor response (86).
NCT02475213 (I)	MGA271 (Enoblituzumab) + Pembrolizumab/MGA012	B7-H3 ADCC-inducing mAb with anti-PD-1 mAb	Melanoma, head and neck cancer, non small cell lung cancer, urothelial carcinoma	Treatment-related AEs grade ≥3 in 28.6% of patients. Objective responses occurred in roughly one third of anti-PD-1/PD-L1-naïve patients (head and neck squamous cell carcinoma and non-small cell lung cancer) (3).
NCT04634825 (II)	Enoblituzumab + Retifanlimab (MGA012) or Tebotelimab (MGD013)	B7-H3 ADCC-inducing mAb with anti-PD-1 mAb (MGA012) or PD-1 X LAG-3 BsAb DART (MGD013)	Head and neck cancer, head and neck neoplasms, head and neck squamous cell carcinoma	Of the MGA271 with MGA012 cohort of 48 patients, 6.3% (3/48) demonstrated a PR, and 41.7% (20/48) demonstrated SD for at least 3 months. Of the MGA271 with MGD013 cohort of 14 patients, 14.3% (2/14) demonstrated a PR, and 35.7% (5/14) demonstrated SD for at least 3 months (Clinicaltrials.gov)
NCT00089245 (I)	^131^I-omburtamab (131 I-8H9)	Radioimmunotherapy	Brain and CNS tumors, neuroblastoma, sarcoma	Of patients with CNS relapse of NB, 44% (7/16) are long-term survivors 13–17 years after compartmental immunotherapy, and only 13% (2/16) experienced a further CNS relapse after receiving compartmental radioimmunotherapy, a marked increase compared to historical data (90).
NCT03275402 (II/III)	^131^I-omburtamab (131 I-8H9)	Radioimmunotherapy	Neuroblastoma, CNS metastases, leptomeningeal metastases	The most frequent grade 3/4 AEs were myelosuppression. Estimated CNS/LM PFS at 6 months was 75%; OS was 79% at 12 months. ORR was 35% (7/20 patients with measurable disease at baseline) with CR of 25%. DCR was 70% (89).
NCT01099644 (I)	^131^I-omburtamab (131 I-8H9)	Radioimmunotherapy	Peritoneal cancer	No dose-limiting toxcities with low radiation exposure to normal organs. Phase II trial initiated (ClinicalTrials.gov identifier: NCT04022213) (124)
NCT00582608 (N/A)	^131^I-omburtamab	Radioimmunotherapy	CNS cancer, neuroblastoma, sarcoma	Demonstrated hepatic uptake despite low level of hepatic expression (124) 80% (4/5) patients were affected/at risk for serious AEs. 100% (5/5) were affected/at risk for other, non-serious AEs. (Clinicaltrials.gov)
NCT01502917 (I)	^124^I-omburtamab	Radioimmunotherapy	Brain cancer, brain stem glioma, DIPG	Five DLTs (5/50). Negligible systemic exposure (91, 96)
NCT04167618 (I/II)	177Lu-DTPA-omburtamab	Radioimmunotherapy	Childhood medulloblastoma	Partial seizures and no non-serious AEs experienced by patient (1/1) administered 10 mCi 177Lu-DTPA-omburtamab. Only non-serious AEs experienced by patient (1/1) administered 25 mCi 177Lu-DTPA-omburtamab (Clinicaltrials.gov)
NCT03729596 (I/II)	MGC018 (vobramitamab duocarmazine) + MGA012	Anti-B7-H3 ADC (MGC018) with anti-PD-1 mAb (MGA012)	Advanced solid tumors	Dose-escalation phase demonstrated evidence of clinical activity in melanoma patients (reductions in target lesion sums). In the cohort expansion phase, TEAEs occurred in 87.7% (43/49) patients. Reductions in target lesion sums were observed in 4/7 evaluable mCRPC patients. There were 11/22 evaluable patients with at least a 50% PSA reduction (125, 126).
NCT04145622 (I/II)	DS-7300a (Ifinatamab Deruxtecan or I-DXd)	ADC	Advanced solid tumors	TEAEs occurred in 98% (124/127) of patients. Responses were observed in 33% (30/91) of evaluable patients in total (7/9 patients with SCLC, 2/5 with sqNSCLC, and 16/42 with mCRPC) Additional findings include ORRs of 25% (15/59) for CRPC, 21% (6/28) for ESCC, 31% (4/13) for sqNSCLC, and 52% (11/21) for SCLC, as well as encouraging DORs and OS (106).
NCT05276609 (I)	HS-20093	ADC	Advanced solid tumors	TEAEs occurred in 53/53 patients. Of response-evaluable patients, 14/40 had PRs (9 confirmed; 5 awaiting confirmation) (35% response rate), and the DCR was 85% (34/40). Response rate in SCLC subset was 77.8% (103)
NCT03595059 (I)	ABBV-155 (Mirzotamab clezutoclax or Mirzo-C) +paclitaxel or docetaxel	ADC	Relapsed and/or refractory solid tumors	Tolerable safety profile, with ORRs of 0% (0/14), 11% (4/36), and 18% (5/28) in the SCLC, NSCLC, and breast cancer cohorts, respectively (127)
NCT05434234 (I) NCT06057922 (I)	YL201	ADC	Advanced solid tumors	Acceptable safety profile and promising efficacy in heavily pre-treated patients. The DCR was 83.6%. Encouraging OR rates included 63.9% in ES-SCLC cancer, 48.6% in nasopharyngeal carcinoma, and 54.2% in lymphoepithelioma-like carcinoma, with an overall OR rate of 40.8% across all patients (75).
CTR20231298 (I/II)	MHB088C	ADC	mCRPC, ES-SCLC	Acceptable safety profile, encouraging efficacy in heavily pretreated mCRPC (14.3% ORR, 95.2% DCR, and 87% rPFS at 6 months), and activity in the 2.0 mg/kg cohort (n=33) of patients with relapsed ES-SCLC (42.4% confirmed ORR, 5.9 months median PFS, and 87.9% DCR) (128, 129)
NCT06008379 (I/II)	7MW3711	ADC	Advanced solid tumors	SCLC patients (n=8) who had previously progressed after other treatment regimens demonstrated an ORR of 62.5% and a DCR of 100% at a dose of 4.5 mg/kg. Encouraging results were also observed in some Sq-NSCLC patients (118)
NCT06008366 (I/II)	7MW3711	ADC	Advanced solid tumors	Patients with advanced esophageal cancer, platinum-resistant ovarian cancer, and prostate cancer, enrolled at a dose of at least 4.5 mg/kg and evaluable for tumor assessment, the ADC demonstrated promising results, with ORRs of 33.3%, 60%, and 50% respectively and DCRs of 100% for all three disease cohorts (n=15) (130).
ChiCTR2100047968	MT027	Off-the-shelf, allogeneic CAR-T (UCAR-T) cells targeting B7-H3	Recurrent high-grade glioma	Acceptable tolerability and safety with 3 cases (6%) of SAEs. After multiple doses, CAR-T cell numbers remained high, and IL6, TNF-α, and IFN-γ levels in CSF increased, with the elevation proving durable. In the cohort (n=15) that received three or more doses and at least one efficacy visit, the ORR was 33%, and the DCR was 80% (119).
NCT04185038 (I)	B7-H3 CAR T cells (SCRI-CARB7H3)	CAR T Cell	CNS tumors, DIPG	In the first three evaluable DIPG, CAR T cells were demonstrated to be able to persist in CSF. Chemokine/cytokine analysis and proteomic assessments support local CNS immune activation. Declining serum levels of B7-H3 were detected throughout treatment. Patients enrolled after initial tumor progression demonstrated increase tumor bulk with infiltration, but the patient who enrolled prior to progression demonstrated a mild decrease in tumor size, reduced conspicuity tumoral nodules, and sustained improvement of facial nerve palsy through 12 months on study (131)
NCT04483778 (I)	B7-H3 CAR T cells (SCRI-CARB7H3) tracking/suicide construct EGFRt + B7-H3/CD19 CAR T cells (SCRI-CARB7H3x19) with tracking/suicide construct HER2tG + prembrolizumab	CAR T Cell/Multi-antigen-targeted CAR T Cell with anti-PD-1 mAb	Pediatric solid tumor	In patients infused with B7-H3 CAR T cells expressing EGFR (Arm A), the best overall response of SD was observed in 3/9 subjects infused. In patients infused with B7-H3xCD19 CAR T cells, the best overall response was observed in 2/11 subjects infused (132)

## Discussion and future perspectives

B7-H3, a member of the B7 family, is an ICP with context-dependent immunoregulatory functions. Although initially described as costimulatory, the preponderance of evidence supports a predominantly coinhibitory role in human cancers, enabling tumor cells to evade immune surveillance.

A defining feature of B7-H3 is its discordant expression pattern, with widespread mRNA expression but restricted protein expression in normal tissues and marked overexpression across diverse malignancies. This tumor-selective protein expression profile provides a strong rationale for therapeutic targeting, with the potential to maximize antitumor efficacy while minimizing off-target toxicity. Furthermore, the consistent association between elevated B7-H3 expression and poor clinical outcomes across multiple cancer types underscores its value not only as a therapeutic target but also as a prognostic biomarker.

Beyond immune modulation, B7-H3 contributes to tumor progression through immune-independent mechanisms, including the promotion of proliferation, metabolic reprogramming, angiogenesis, and metastasis. These multifaceted roles further reinforce its relevance as a central regulator of tumor biology and a compelling candidate for targeted intervention. Emerging evidence also highlights the importance of molecular heterogeneity in B7-H3 biology. Differential expression of the 2Ig and 4Ig isoforms, as well as post-translational modifications such as glycosylation, may influence functional activity, immune interactions, and therapeutic susceptibility. Similarly, the potential roles of B7-H3 dimerization and cis-signaling mechanisms remain poorly understood and warrant further investigation. Epigenetic and post-transcriptional regulatory mechanisms, including DNA methylation, histone modifications, and miRNA-mediated control, represent additional layers of complexity that may offer novel therapeutic entry points. Despite substantial progress in therapeutic development, several critical knowledge gaps remain. The absence of a definitively validated receptor continues to limit mechanistic understanding and hinders the rational design of next-generation therapies. In addition, the signaling pathways downstream of B7-H3 remain incompletely defined and may vary across tumor types and cellular contexts. Addressing these gaps will be essential for optimizing therapeutic strategies and predicting treatment response. Clinically, B7-H3-directed therapies have demonstrated encouraging early-phase results across multiple malignancies. While no agents have yet received FDA approval, the recent Breakthrough Therapy Designation granted to ifinatamab deruxtecan (I-DXd) highlights the accelerating clinical momentum in this space. However, key challenges remain, including the identification of predictive biomarkers, optimization of patient selection, and management of treatment-related toxicities. Despite the promising therapeutic advances targeting B7-H3, variability in clinical responses remains a major challenge. Differences in B7-H3 expression levels, together with inter- and intra-tumoral heterogeneity, may contribute to inconsistent treatment efficacy across patients and tumor types. Furthermore, reliance on immunohistochemistry-based assessment of tumor biopsies is limited by sampling bias and may not fully capture the spatial and temporal heterogeneity of antigen expression. Emerging non-invasive molecular imaging approaches, including radiolabeled antibodies and positron emission tomography-based strategies, may provide a more comprehensive and dynamic assessment of B7-H3 expression. These techniques could improve patient stratification, facilitate treatment monitoring, and help identify patients most likely to benefit from B7-H3-targeted therapies.

## Conclusion

In summary, B7-H3 represents a promising and versatile target at the intersection of tumor immunology and cancer cell-intrinsic signaling. Continued integration of mechanistic insights with clinical investigation will be critical to fully realizing the therapeutic potential of B7-H3-targeted strategies and translating this promise into durable clinical benefit for patients.

## References

[1] He X Xu C . Immune checkpoint signaling and cancer immunotherapy. Cell Res. (2020) 30:660–9. doi: 10.1038/s41422-020-0343-4 32467592 PMC7395714

[2] Sharma P Goswami S Raychaudhuri D Siddiqui BA Singh P Nagarajan A . Immune checkpoint therapy—current perspectives and future directions. Cell. (2023) 186:1652–69. doi: 10.1016/j.cell.2023.03.006 37059068

[3] Gaikwad S Agrawal MY Kaushik I Ramachandran S Srivastava SK . Immune checkpoint proteins: Signaling mechanisms and molecular interactions in cancer immunotherapy. Semin Cancer Biol. (2022) 86:137–50. doi: 10.1016/j.semcancer.2022.03.014 35341913

[4] Postow MA Sidlow R Hellmann MD . Immune-related adverse events associated with immune checkpoint blockade. N Engl J Med. (2018) 378:158–68. doi: 10.1056/nejmra1703481 29320654

[5] Sharma P Hu-Lieskovan S Wargo JA Ribas A . Primary, adaptive, and acquired resistance to cancer immunotherapy. Cell. (2017) 168:707–23. doi: 10.1016/j.cell.2017.01.017 28187290 PMC5391692

[6] Ribas A Wolchok JD . Cancer immunotherapy using checkpoint blockade. Science. (2018) 359:1350–5. doi: 10.1126/science.aar4060 29567705 PMC7391259

[7] Bretscher P Cohn M . A theory of self-nonself discrimination. Sci (American Assoc For Advancement Science). (1970) 169:1042–9. doi: 10.1126/science.169.3950.1042 4194660

[8] Collins M Ling V Carreno BM . The B7 family of immune-regulatory ligands. Genome Biol. (2005) 6:223. 15960813 10.1186/gb-2005-6-6-223 PMC1175965

[9] Sharpe AH Freeman GJ . The B7–CD28 superfamily. Nat Rev Immunol. (2002) 2:116–26. 10.1038/nri727 11910893

[10] Greaves P Gribben JG . The role of B7 family molecules in hematologic Malignancy. Blood. (2013) 121:734–44. doi: 10.1182/blood-2012-10-385591 23223433 PMC3563361

[11] Vigdorovich V Ramagopal A Udupi Lázár-Molnár E Sylvestre E Lee S Jun Hofmeyer A . Structure and T cell inhibition properties of B7 family member, B7-H3. Structure. (2013) 21:707–17. doi: 10.1016/j.str.2013.03.003 23583036 PMC3998375

[12] Chapoval AI Ni J Lau JS Wilcox RA Flies DB Liu D . B7-H3: A costimulatory molecule for T cell activation and IFN-γ production. Nat Immunol. (2001) 2:269–74. doi: 10.1038/85339 11224528

[13] Tyagi A Ly S El-Dana F Yuan B Jaggupilli A Grimm S . Evidence supporting a role for the immune checkpoint protein B7-H3 in NK cell-mediated cytotoxicity against AML. Blood. (2022) 139:2782–96. doi: 10.1182/blood.2021014671 35231101 PMC11022957

[14] Bork P Holm L Sander C . The immunoglobulin fold. J Mol Biol. (1994) 242:309–20. doi: 10.1006/jmbi.1994.1582 7932691

[15] Sun M Richards S Prasad DVR Mai XM Rudensky A Dong C . Characterization of mouse and human B7-H3 genes. J Immunol. (2002) 168:6294–7. doi: 10.4049/jimmunol.168.12.6294 12055244

[16] Zhou YH Chen YJ Ma ZY Xu L Wang Q Zhang GB . 4IgB7‐H3 is the major isoform expressed on immunocytes as well as Malignant cells. Tissue Antigens. (2007) 70:96–104. doi: 10.1111/j.1399-0039.2007.00853.x 17610414

[17] Chapoval AI Ni J Lau JS Wilcox RA Flies DB Liu D . B7-H3: a costimulatory molecule for T cell activation and IFN-gamma production. Nat Immunol. (2001) 2:269–74. doi: 10.1038/85339 11224528

[18] Kontos F Michelakos T Kurokawa T Sadagopan A Schwab JH Ferrone CR . B7-H3: An attractive target for antibody-based immunotherapy. Clin Cancer Res. (2021) 27:1227–35. doi: 10.1158/1078-0432.ccr-20-2584 33051306 PMC7925343

[19] Zekri L Lutz M Prakash N Manz T Klimovich B Mueller S . An optimized IgG-based B7-H3xCD3 bispecific antibody for treatment of gastrointestinal cancers. Mol Ther. (2023) 31:1033–45. doi: 10.1016/j.ymthe.2023.02.010 36793213 PMC10124076

[20] Zhang G Hou J Shi J Yu G Lu B Zhang X . Soluble CD276 (B7‐H3) is released from monocytes, dendritic cells and activated T cells and is detectable in normal human serum. Immunology. (2008) 123:538–46. doi: 10.1111/j.1365-2567.2007.02723.x 18194267 PMC2433324

[21] Huang L Zhao Y Sun Q Cao L Zhang X . Evaluation of the role of soluble B7-H3 in association with membrane B7-H3 expression in gastric adenocarcinoma - PubMed. Cancer Biomarkers: Section A Dis Markers. (2022) 33(1):123–9. doi: 10.3233/cbm-210178 34459388 PMC12364134

[22] Chen W Liu P Wang Y Nie W Li Z Xu W . Characterization of a soluble B7-H3 (sB7-H3) spliced from the intron and analysis of sB7-H3 in the sera of patients with hepatocellular carcinoma. PloS One. (2013) 8:e76965. doi: 10.1371/journal.pone.0076965 24194851 PMC3806749

[23] Zhao Y Sun K Yu Y Xu J Wang Y Yang C . sB7-H3 as a prognostic biomarker in osteosarcoma: Insights into clinical outcomes. Sci Rep. (2026) 16(1):10169. doi: 10.1038/s41598-026-40855-2 41724790 PMC13022227

[24] Azuma T Sato Y Ohno T Azuma M Kume H . Serum soluble B7-H3 is a prognostic marker for patients with non-muscle-invasive bladder cancer. PloS One. (2020) 15:e0243379. 33306717 10.1371/journal.pone.0243379 PMC7732087

[25] Glazer SE Sutton MN Yang P Pisaneschi F Sebastian M Gammon ST . Anti-cancer immune priming with β-radioligand therapy using a novel high affinity antibody selectively targeting the 4Ig-isoform of B7-H3. Theranostics. (2026) 16:5370–92. doi: 10.7150/thno.123285 41993624 PMC13080693

[26] Sutton MN Glazer SE Muzzioli R Yang P Gammon ST Piwnica-Worms D . Dimerization of the 4Ig isoform of B7-H3 in tumor cells mediates enhanced proliferation and tumorigenic signaling. Commun Biol. (2024) 7(1):21. doi: 10.1038/s42003-023-05736-8 38182652 PMC10770396

[27] Ford JW McVicar DW . TREM and TREM-like receptors in inflammation and disease. Curr Opin Immunol. (2009) 21:38–46. doi: 10.1016/j.coi.2009.01.009 19230638 PMC2723941

[28] Halpert MM Thomas KA King RG Justement LB . TLT2 potentiates neutrophil antibacterial activity and chemotaxis in response to G protein-coupled receptor-mediated signaling. J Immunol. (2011) 187:2346–55. doi: 10.4049/jimmunol.1100534 21804015 PMC3159717

[29] Hashiguchi M Kobori H Ritprajak P Kamimura Y Kozono H Azuma M . Triggering receptor expressed on myeloid cell-like transcript 2 (TLT-2) is a counter-receptor for B7-H3 and enhances T cell responses. Proc Natl Acad Sci. (2008) 105:10495–500. doi: 10.1073/pnas.0802423105 18650384 PMC2492502

[30] Leitner J Klauser C Pickl WF Stöckl J Majdic O Bardet AF . B7‐H3 is a potent inhibitor of human T‐cell activation: No evidence for B7‐H3 and TREML2 interaction. Eur J Immunol. (2009) 39:1754–64. doi: 10.1002/eji.200839028 19544488 PMC2978551

[31] Husain B Ramani SR Chiang E Lehoux I Paduchuri S Arena TA . A platform for extracellular interactome discovery identifies novel functional binding partners for the immune receptors B7-H3/CD276 and PVR/CD155. Mol Cell Proteomics. (2019) 18:2310–23. doi: 10.1093/annonc/mdz451.008 PMC6823854 31308249

[32] Gao W Wen H Liang L Dong X Du R Zhou W . IL20RA signaling enhances stemness and promotes the formation of an immunosuppressive microenvironment in breast cancer. Theranostics. (2021) 11:2564–80. doi: 10.7150/thno.45280 33456560 PMC7806486

[33] Yu D Yang X Lin J Cao Z Lu C Yang Z . Super-enhancer induced IL-20RA promotes proliferation/metastasis and immune evasion in colorectal cancer. Front Oncol. (2021) 11. doi: 10.3389/fonc.2021.724655 34336707 PMC8319729

[34] You G Lee Y Kang Y-W Park HW Park K Kim H . B7-H3×4-1BB bispecific antibody augments antitumor immunity by enhancing terminally differentiated CD8^+^ tumor-infiltrating lymphocytes. Sci Adv. (2021) 7:eaax3160. doi: 10.1126/sciadv.aax3160 33523913 PMC7810375

[35] Bernard D Vindrieux D . PLA2R1: Expression and function in cancer. Biochim Biophys Acta. (2014) 1846:40–4. doi: 10.1016/j.bbcan.2014.03.003 24667060

[36] Huna A Griveau A Vindrieux D Jaber S Flaman J-M Goehrig D . PLA2R1 promotes DNA damage and inhibits spontaneous tumor formation during aging. Cell Death Dis. (2021) 12(2):190. doi: 10.1038/s41419-021-03468-3 33594040 PMC7887270

[37] Cao S Peterson SM Müller S Reichelt M Mcroberts Amador C Martinez-Martin N . A membrane protein display platform for receptor interactome discovery. Proc Natl Acad Sci. (2021) 118:e2025451118. doi: 10.1073/pnas.2025451118 34531301 PMC8488672

[38] Ciprut S Berberich A Knoll M Pusch S Hoffmann D Furkel J . AAMP is a binding partner of costimulatory human B7-H3. Neuro-Oncology Adv. (2022) 4(1):vdac098. doi: 10.1093/noajnl/vdac098 35919070 PMC9341442

[39] Hofmeyer KA Ray A Zang X . The contrasting role of B7-H3. Proc Natl Acad Sci. (2008) 105:10277–8. doi: 10.1073/pnas.0805458105 18650376 PMC2492485

[40] Liu H-J Du H Khabibullin D Zarei M Wei K Freeman GJ . mTORC1 upregulates B7-H3/CD276 to inhibit antitumor T cells and drive tumor immune evasion. Nat Commun. (2023) 14(1):1214. doi: 10.1038/s41467-023-36881-7 36869048 PMC9984496

[41] Zhang C Chen Y Li F Yang M Meng F Zhang Y . B7-H3 is spliced by SRSF3 in colorectal cancer. Cancer Immunol Immunother. (2021) 70:311–21. doi: 10.1007/s00262-020-02683-9 32719950 PMC10991627

[42] Chen R Su F Zhang T Wu D Yang J Guan Q . N6-methyladenosine modification of B7-H3 mRNA promotes the development and progression of colorectal cancer. iScience. (2024) 27:108956. doi: 10.1016/j.isci.2024.108956 38318386 PMC10839442

[43] Flem-Karlsen K Fodstad Ø Tan M Nunes-Xavier CE . B7-H3 in cancer – beyond immune regulation. Trends Cancer. (2018) 4:401–4. doi: 10.1016/j.trecan.2018.03.010 29860983

[44] Xu H Cheung IY Guo H-F Cheung N-KV . MicroRNA miR-29 modulates expression of immunoinhibitory molecule B7-H3: Potential implications for immune based therapy of human solid tumors. Cancer Res. (2009) 69:6275–81. doi: 10.1158/0008-5472.can-08-4517 19584290 PMC2719680

[45] Modak S Kramer K Gultekin SH Guo HF Cheung N-KV . Monoclonal antibody 8H9 targets a novel cell surface antigen expressed by a wide spectrum of human solid tumors1. Cancer Res. (2001) 61:4048–54. 11358824

[46] Zhao B Li H Xia Y Wang Y Wang Y Shi Y . Immune checkpoint of B7-H3 in cancer: From immunology to clinical immunotherapy. J Hematol Oncol. (2022) 15:153. doi: 10.1186/s13045-022-01364-7 36284349 PMC9597993

[47] Pathania AS Chava H Chaturvedi NK Chava S Byrareddy SN Coulter DW . The miR-29 family facilitates the activation of NK-cell immune responses by targeting the B7-H3 immune checkpoint in neuroblastoma. Cell Death Dis. (2024) 15:428. doi: 10.1038/s41419-024-06791-7 38890285 PMC11189583

[48] Xie Y Wang H Zeng F Zhang Y Huang J Chen C . Exploiting B7-H3: Molecular insights and immunotherapeutic strategies for osteosarcoma. Bioengineering (Basel). (2025) 12(12):1344. doi: 10.3390/bioengineering12121344 41463642 PMC12729273

[49] Castriconi R Dondero A Augugliaro R Cantoni C Carnemolla B Sementa AR . Identification of 4Ig-B7-H3 as a neuroblastoma-associated molecule that exerts a protective role from an NK cell-mediated lysis. Proc Natl Acad Sci. (2004) 101:12640–5. doi: 10.1073/pnas.0405025101 15314238 PMC515110

[50] Huang Y Zhang H-L Li Z-L Du T Chen Y-H Wang Y . FUT8-mediated aberrant N-glycosylation of B7H3 suppresses the immune response in triple-negative breast cancer. Nat Commun. (2021) 12(1):2672. doi: 10.1038/s41467-021-22618-x 33976130 PMC8113546

[51] Chen J-T Chen C-H Ku K-L Hsiao M Chiang C-P Hsu T-L . Glycoprotein B7-H3 overexpression and aberrant glycosylation in oral cancer and immune response. Proc Natl Acad Sci. (2015) 112:13057–62. doi: 10.1073/pnas.1516991112 26438868 PMC4620862

[52] Luo L Chapoval AI Flies DB Zhu G Hirano F Wang S . B7-H3 enhances tumor immunity *in vivo* by costimulating rapid clonal expansion of antigen-specific CD8+ cytolytic T cells. J Immunol. (2004) 173:5445–50. doi: 10.4049/jimmunol.173.9.5445 15494491

[53] Suh W-K Gajewska BU Okada H Gronski MA Bertram EM Dawicki W . The B7 family member B7-H3 preferentially down-regulates T helper type 1–mediated immune responses. Nat Immunol. (2003) 4:899–906. doi: 10.1038/ni967 12925852

[54] Lee Y-H Martin-Orozco N Zheng P Li J Zhang P Tan H . Inhibition of the B7-H3 immune checkpoint limits tumor growth by enhancing cytotoxic lymphocyte function. Cell Res. (2017) 27:1034–45. doi: 10.1038/cr.2017.90 28685773 PMC5539354

[55] Lim S Liu H Da Silva LM Arora R Liu Z Phillips JB . Immunoregulatory protein B7-H3 reprograms glucose metabolism in cancer cells by ROS-mediated stabilization of HIF1α. Cancer Res. (2016) 76:2231–42. doi: 10.1158/0008-5472.can-15-1538 27197253 PMC4874665

[56] Mao Y Chen L Wang F Zhu D Ge X Hua D . Cancer cell-expressed B7-H3 regulates the differentiation of tumor-associated macrophages in human colorectal carcinoma. Oncol Lett. (2017) 14(5):6177–83. doi: 10.3892/ol.2017.6935 29113264 PMC5661406

[57] Wang S Wang J Chen Z Luo J Guo W Sun L . Targeting M2-like tumor-associated macrophages is a potential therapeutic approach to overcome antitumor drug resistance. NPJ Precis Oncol. (2024) 8(1):31. doi: 10.1038/s41698-024-00522-z 38341519 PMC10858952

[58] Zhang LY Jin Y Xia PH Lin J Ma JC Li T . Integrated analysis reveals distinct molecular, clinical, and immunological features of B7‐H3 in acute myeloid leukemia. Cancer Med. (2021) 10:7831–46. doi: 10.1002/cam4.4284 34562306 PMC8559480

[59] Miyamoto T Murakami R Hamanishi J Tanigaki K Hosoe Y Mise N . B7-H3 suppresses antitumor immunity via the CCL2–CCR2–M2 macrophage axis and contributes to ovarian cancer progression. Cancer Immunol Res. (2022) 10:56–69. doi: 10.1158/2326-6066.cir-21-0407 34799346 PMC9414298

[60] Hanahan D . Hallmarks of cancer: New dimensions. Cancer Discov. (2022) 12:31–46. doi: 10.1158/2159-8290.cd-21-1059 35022204

[61] Lin L Cao L Liu Y Wang K Zhang X Qin X . B7-H3 promotes multiple myeloma cell survival and proliferation by ROS-dependent activation of Src/STAT3 and c-Cbl-mediated degradation of SOCS3. Leukemia. (2019) 33:1475–86. doi: 10.1038/s41375-018-0331-6 30573782

[62] Liu J Yang S Cao B Zhou G Zhang F Wang Y . Targeting B7-H3 via chimeric antigen receptor T cells and bispecific killer cell engagers augments antitumor response of cytotoxic lymphocytes. J Hematol Oncol. (2021) 14(1):21. doi: 10.1186/s13045-020-01024-8 33514401 PMC7844995

[63] Liu H Tekle C Chen Y-W Kristian A Zhao Y Zhou M . B7-H3 silencing increases paclitaxel sensitivity by abrogating Jak2/Stat3 phosphorylation. Mol Cancer Ther. (2011) 10:960–71. doi: 10.1158/1535-7163.mct-11-0072 21518725 PMC3253760

[64] Zhou L Zhao Y . &lt;p&gt;B7-H3 induces ovarian cancer drugs resistance through an PI3K/AKT/BCL-2 signaling pathway&lt;/p&gt;. Cancer Manage Res. (2019) 11:10205–14. doi: 10.2147/cmar.s222224 31819652 PMC6899073

[65] Wang R Ma Y Zhan S Zhang G Cao L Zhang X . B7-H3 promotes colorectal cancer angiogenesis through activating the NF-κB pathway to induce VEGFA expression. Cell Death Dis. (2020) 11(1):55. doi: 10.2139/ssrn.3449325 31974361 PMC6978425

[66] Wu R Zhang Y Xu X You Q Yu C Wang W . Exosomal B7-H3 facilitates colorectal cancer angiogenesis and metastasis through AKT1/mTOR/VEGFA pathway. Cell Signal. (2023) 109:110737. doi: 10.1016/j.cellsig.2023.110737 37263461

[67] Lai H Sun Z Yang J Wu P Guo Y Sun J . B7-H3 modulates endothelial cell angiogenesis through the VEGF cytokine. Immunol Res. (2019) 67:202–11. doi: 10.1007/s12026-019-09084-w 31292886

[68] Miller CD Lozada JR Zorko NA Elliott A Makovec A Radovich M . Pan-cancer interrogation of B7-H3 (CD276) as an actionable therapeutic target across human Malignancies. Cancer Res Commun. (2024) 4:1369–79. doi: 10.1158/2767-9764.crc-23-0546 38709075 PMC11138391

[69] Kang F-B Wang L Jia H-C Li D Li H-J Zhang Y-G . B7-H3 promotes aggression and invasion of hepatocellular carcinoma by targeting epithelial-to-mesenchymal transition via JAK2/STAT3/Slug signaling pathway. Cancer Cell Int. (2015) 15(1):45. doi: 10.1186/s12935-015-0195-z 25908926 PMC4407415

[70] Zhou L Zhao Y . B7-H3 induces ovarian cancer drugs resistance through an PI3K/AKT/BCL-2 signaling pathway. Cancer Manag Res. (2019) 11:10205–14. doi: 10.2147/cmar.s222224 31819652 PMC6899073

[71] Yang Q Cao W Wang Z Zhang B Liu J . Regulation of cancer immune escape: The roles of miRNAs in immune checkpoint proteins. Cancer Lett. (2018) 431:73–84. doi: 10.1016/j.canlet.2018.05.015 29800685

[72] Zhang C Zhang Z Li F Shen Z Qiao Y Li L . Large-scale analysis reveals the specific clinical and immune features of B7-H3 in glioma. OncoImmunology. (2018) 7:e1461304. doi: 10.1080/2162402x.2018.1461304 30377558 PMC6205005

[73] Wang Z Wang Z Zhang C Liu X Li G Liu S . Genetic and clinical characterization of B7‐H3 (CD276) expression and epigenetic regulation in diffuse brain glioma. Cancer Sci. (2018) 109:2697–705. doi: 10.1111/cas.13744 30027617 PMC6125452

[74] Chen Y Wu Y Yang H Wang J Kong J Yu L . DNA methylation and mRNA expression of B7-H3 gene in ankylosing spondylitis: A case-control study. Immunol Invest. (2022) 51:2025–34. doi: 10.1080/08820139.2022.2095285 35786112

[75] Ma Y Yang Y Huang Y Fang W Xue J Meng X . A B7H3-targeting antibody–drug conjugate in advanced solid tumors: a phase 1/1b trial. Nat Med. (2025) 31:1949–57. doi: 10.1038/s41591-025-03600-2 40082695 PMC12176648

[76] Zhang D Tang Z Huang H Zhou G Cui C Weng Y . Metabolic regulation of gene expression by histone lactylation. Nature. (2019) 574:575–80. doi: 10.1038/s41586-019-1678-1 31645732 PMC6818755

[77] Costa PMDS Sales SLA Pinheiro DP Pontes LQ Maranhão SSA Pessoa CDÓ . Epigenetic reprogramming in cancer: From diagnosis to treatment. Front Cell Dev Biol. (2023) 11:1116805. doi: 10.3389/fcell.2023.1116805 36866275 PMC9974167

[78] Anand V Tyagi A Battula VL . Abstract P2-20-06: Anti-B7-H3 antibody (T-1A5) blocks immunomodulatory function of B7-H3 and enhances NK and T cell–mediated cytotoxicity against breast cancer cells. Cancer Res. (2023) 83(5_Supplement). doi: 10.1158/1538-7445.sabcs22-p2-20-06 36230740

[79] Wang Z Yang J Zhu Y Zhu Y Zhang B Zhou Y . Differential expression of 2IgB7-H3 and 4IgB7-H3 in cancer cell lines and glioma tissues. Oncol Lett. (2015) 10:2204–8. doi: 10.3892/ol.2015.3611 26622819 PMC4579917

[80] Zhang W Zhang L Qian J Lin J Chen Q Yuan Q . Expression characteristic of 4Ig B7-H3 and 2Ig B7-H3 in acute myeloid leukemia. Bioengineered. (2021) 12. doi: 10.3389/fendo.2024.1481649 34787059 PMC8810086

[81] Mortezaee K . B7-H3 immunoregulatory roles in cancer. Biomedicine pharmacotherapy. (2023) 163:114890. doi: 10.1016/j.biopha.2023.114890 37196544

[82] Zang X Thompson RH Al-Ahmadie HA Serio AM Reuter VE Eastham JA . B7-H3 and B7x are highly expressed in human prostate cancer and associated with disease spread and poor outcome. Proc Natl Acad Sci. (2007) 104:19458–63. doi: 10.1016/s0022-5347(08)60301-8 PMC2148311 18042703

[83] Amori G Sugawara E Shigematsu Y Akiya M Kunieda J Yuasa T . Tumor B7-H3 expression in diagnostic biopsy specimens and survival in patients with metastatic prostate cancer. Prostate Cancer Prostatic Dis. (2021) 24:767–74. doi: 10.1038/s41391-021-00331-6 33558663

[84] Ahmed M Cheng M Zhao Q Goldgur Y Cheal SM Guo H-F . Humanized affinity-matured monoclonal antibody 8H9 has potent antitumor activity and binds to FG loop of tumor antigen B7-H3 *. J Biol Chem. (2015) 290(50):30018–29. doi: 10.1074/jbc.m115.679852 26487718 PMC4705981

[85] Loo D Alderson RF Chen FZ Huang L Zhang W Gorlatov S . Development of an Fc-enhanced anti–B7-H3 monoclonal antibody with potent antitumor activity. Clin Cancer Res. (2012) 18:3834–45. doi: 10.1158/1078-0432.ccr-12-0715 22615450

[86] Shenderov E De Marzo AM Lotan TL Wang H Chan S Lim SJ . Neoadjuvant enoblituzumab in localized prostate cancer: a single-arm, phase 2 trial. Nat Med. (2023) 29:888–97. doi: 10.21417/es2023nm PMC10921422 37012549

[87] Aggarwal C Prawira A Antonia S Rahma O Tolcher A Cohen RB . Dual checkpoint targeting of B7-H3 and PD-1 with enoblituzumab and pembrolizumab in advanced solid tumors: interim results from a multicenter phase I/II trial. J Immunother Cancer. (2022) 10(4):e004424. doi: 10.1136/jitc-2021-004424 35414591 PMC9006844

[88] Nagase‐Zembutsu A Hirotani K Yamato M Yamaguchi J Takata T Yoshida M . Development of <scp>DS</scp>‐5573a: A novel afucosylated <scp>mA</scp>b directed at B7‐H3 with potent antitumor activity. Cancer Sci. (2016) 107:674–81. 10.1111/cas.12915 PMC4970835 26914241

[89] Basu E Mora J Streby K Bear M Sano H Marachelian A . LBA3 compartmental radioimmunotherapy (cRIT) 131I-OMBURTAMAB in patients with neuroblastoma (NB) central nervous system (CNS) and/or leptomeningeal (LM) metastases: Updated results from pivotal trial 101. Immuno-Oncology Technol. (2022) 16:100364. doi: 10.1016/j.iotech.2022.100364 38826717

[90] Kramer K Pandit-Taskar N Kushner BH Zanzonico P Humm JL Tomlinson U . Phase 1 study of intraventricular 131I-omburtamab targeting B7H3 (CD276)-expressing CNS Malignancies. J Hematol Oncol. (2022) 15(1):165. doi: 10.1186/s13045-022-01383-4 36371226 PMC9655863

[91] Souweidane MM Kramer K Pandit-Taskar N Zhou Z Haque S Zanzonico P . Convection-enhanced delivery for diffuse intrinsic pontine glioma: a single-centre, dose-escalation, phase 1 trial. Lancet Oncol. (2018) 19:1040–50. doi: 10.1016/s1470-2045(18)30322-x 29914796 PMC6692905

[92] Kasten BB Arend RC Katre AA Kim H Fan J Ferrone S . B7-H3-targeted 212Pb radioimmunotherapy of ovarian cancer in preclinical models. Nucl Med Biol. (2017) 47:23–30. doi: 10.1016/j.nucmedbio.2017.01.003 28104527 PMC5340614

[93] Wang G Wu Z Wang Y Li X Zhang G Hou J . Therapy to target renal cell carcinoma using 131I-labeled B7-H3 monoclonal antibody. Oncotarget. (2016) 7:24888–98. doi: 10.18632/oncotarget.8550 27058890 PMC5029751

[94] Kasten BB Gangrade A Kim H Fan J Ferrone S Ferrone CR . 212Pb-labeled B7-H3-targeting antibody for pancreatic cancer therapy in mouse models. Nucl Med Biol. (2018) 58:67–73. doi: 10.1016/j.nucmedbio.2017.12.004 29413459 PMC5817013

[95] Erratum to: Phase 1 dose-escalation trial using convection-enhanced delivery of radio-immunotheranostic 124I-Omburtamab for diffuse intrinsic pontine glioma. Neuro Oncol. (2026) 28:e10. 40489365 10.1093/neuonc/noaf138 PMC13128442

[96] Souweidane MM Bander ED Zanzonico P Reiner AS Manino N Haque S . Phase 1 dose-escalation trial using convection-enhanced delivery of radio-immunotheranostic 124I-Omburtamab for diffuse intrinsic pontine glioma. Neuro Oncol. (2025) 27:2117–26. 10.1093/neuonc/noaf039 PMC12448826 39969230

[97] Meeus F Funeh CN Awad RM Zeven K Autaers D De Becker A . Preclinical evaluation of antigen-sensitive B7-H3-targeting nanobody-based CAR-T cells in glioblastoma cautions for on-target, off-tumor toxicity. J Immunother Cancer. (2024) 12(11):e009110. 39562005 10.1136/jitc-2024-009110 PMC11575280

[98] Oroujeni M Bezverkhniaia EA Xu T Liu Y Plotnikov EV Karlberg I . Evaluation of an affibody-based binder for imaging of immune check-point molecule B7-H3. Pharmaceutics. (2022) 14(9):1780. 36145529 10.3390/pharmaceutics14091780 PMC9506244

[99] Tolmachev V Bezverkhniaia EA Papalanis E Muhammad AMB Vorobyeva A Gunneriusson E . Preclinical positron emission tomography imaging of B7-H3 expression using affibody molecules labeled with gallium-68. ACS Pharmacol Transl Sci. (2025) 8:3509–22. 10.1021/acsptsci.5c00320 PMC12519266 41098568

[100] Scribner JA Brown JG Son T Chiechi M Li P Sharma S . Preclinical development of MGC018, a duocarmycin-based antibody–drug conjugate targeting B7-H3 for solid cancer. Mol Cancer Ther. (2020) 19:2235–44. 10.1158/1535-7163.MCT-20-0116 32967924

[101] Brignole C Calarco E Bensa V Giusto E Perri P Ciampi E . Antitumor activity of the investigational B7-H3 antibody-drug conjugate, vobramitamab duocarmazine, in preclinical models of neuroblastoma. J Immunother Cancer. (2023) 11:e007174. 37775116 10.1136/jitc-2023-007174 PMC10546160

[102] Bianchi G Pastorino F Rolandi G Ciampi E Segalerba D De Giovanni B . The investigational anti-B7-H3 antibody-drug conjugate vobramitamab duocarmazine exerts anti-tumor activity *in vitro* and *in vivo* in pediatric sarcoma preclinical models. Cell Death Dis. (2026) (1):173. 41507126 10.1038/s41419-025-08397-z PMC12877178

[103] Wang J Duan J Xing L Sun Y Guo W Wang H . ARTEMIS-001: Phase 1 study of HS-20093, a B7-H3–targeting antibody-drug conjugate, in patients with advanced solid tumor. J Clin Oncol. (2023) 41:3017.

[104] Wang Z Ramage CL Ma H Zhang H Souers A Phillips DC . Anti-leukemia combinatorial efficacy of ABBV155, a B7H3-BCL-XL inhibitor antibody-drug conjugate, in combination with venetoclax through BCL-XL/BIM and BCL-2/BIM complex dissociations. Blood. (2024) 144:2760.

[105] Yamato M Hasegawa J Maejima T Hattori C Kumagai K Watanabe A . DS-7300a, a DNA topoisomerase I inhibitor, DXd-based antibody–drug conjugate targeting B7-H3, exerts potent antitumor activities in preclinical models. Mol Cancer Ther. (2022) 21:635–46. 10.1158/1535-7163.MCT-21-0554 PMC9377751 35149548

[106] Patel MR Doi T Koyama T Falchook GS Friedman CF Piha-Paul SA . 690P Ifinatamab deruxtecan (I-DXd; DS-7300) in patients with advanced solid tumors: Updated clinical and biomarker results from a phase I/II study. Ann Oncol. (2023) 34:S481–2.

[107] Owonikoko TK Byers L Cheng Y Hayashi H Paz-Ares L Pérol M . IDeate-Lung02: a Phase 3 study of second-line ifinatamab deruxtecan in patients with relapsed small cell lung cancer. Future Oncol. (2025) 21:3275–82. 10.1080/14796694.2025.2565995 PMC12536792 41055143

[108] Doi T Patel M Falchook GS Koyama T Friedman CF Piha-Paul S . 453O DS-7300 (B7-H3 DXd antibody-drug conjugate [ADC]) shows durable antitumor activity in advanced solid tumors: Extended follow-up of a phase I/II study. Ann Oncol. (2022) 33:S744–5.

[109] Rudin CM Johnson ML Paz-Ares L Nishio M Hann CL Girard N . Ifinatamab deruxtecan in patients with extensive-stage small cell lung cancer: primary analysis of the phase II IDeate-lung01 trial. J Clin Oncol. (2026) 44(4):261–73. 10.1200/JCO-25-02142 PMC12834294 41086386

[110] Li Z Wang Q Han L Ouyang W Li X Yao Y . Results from a phase 1/2 study of 7MW3711: A novel B7-H3 antibody-drug conjugate (ADC) incorporating a topoisomerase I inhibitor in patients with lung cancer. J Clin Oncol. (2025) 43(16_suppl).

[111] Menon AP Moreno B Meraviglia-Crivelli D Nonatelli F Villanueva H Barainka M . Modulating T cell responses by targeting CD3. Cancers. (2023) 15:1189. 36831533 10.3390/cancers15041189 PMC9953819

[112] Shankar S Spira AI Strauss J Liu L La Motte-Mohs R Wu T . A phase 1, open label, dose escalation study of MGD009, a humanized B7-H3 x CD3 DART protein, in combination with MGA012, an anti-PD-1 antibody, in patients with relapsed or refractory B7-H3-expressing tumors. J Clin Oncol. (2018) 36:TPS2601–TPS2601.

[113] Feng Y Xie K Yin Y Li B Pi C Xu X . A novel anti-B7-H3 × Anti-CD3 bispecific antibody with potent antitumor activity. Life. (2022) 12:157. 35207448 10.3390/life12020157 PMC8879513

[114] Mews EA Beckmann P Patchava M Wang Y Largaespada DA Wagner CR . Multivalent, bispecific αB7-H3-αCD3 chemically self-assembled nanorings direct potent T cell responses against medulloblastoma. ACS Nano. (2022) 16(8):12185–201. 10.1021/acsnano.2c02850 PMC9885520 35876221

[115] Li H-Y Chen Y-L Deng X-N Li H-H Tan J Liu G-J . Bispecific antibody targeting both B7-H3 and PD-L1 exhibits superior antitumor activities. Acta Pharmacol Sin. (2023) 44:2322–30. 10.1038/s41401-023-01118-2 PMC10618207 37328649

[116] Guan J Zhou S Wu W Zhu T Cao L Wu M . Abstract LB056: IBI334, a novel ADCC-enhanced B7-H3/EGFR bispecific antibody, demonstrated potent pre-clinical efficacy in solid tumors. Cancer Res. (2024) 84(7_Supplement).

[117] Zhi X Wang J Guo J Luo L Sun H Li Y . Development of a bispecific antibody that inhibits EGFR and B7H3 in NSCLC. biomark Res. (2025) 13(1):158. 41291854 10.1186/s40364-025-00872-1 PMC12750570

[118] Qi L Takeda S Zhang Y Huang M Zhou D Mao Y . Abstract 2955: Preclinical development of TJ101, a potent bispecific ADC targeting EGFR and B7-H3 for the treatment of solid cancers. Cancer Res. (2025) 85(8_Supplement_1).

[119] Shang Y Qiu J Xin Y Zhao M Liu Q Wang Z . Abstract 5087: ILB-3101, a novel B7H3-targeting ADC with Eribulin as payload, demonstrates strong tumor killing activities and favorable PK/TOX profile in preclinical evaluation. Cancer Res. (2024) 84(6_Supplement).

[120] Yang S Cao B Zhou G Zhu L Wang L Zhang L . Targeting B7-H3 immune checkpoint with chimeric antigen receptor-engineered natural killer cells exhibits potent cytotoxicity against non-small cell lung cancer. Front Pharmacol. (2020) 11. 10.3389/fphar.2020.01089 PMC7406658 32848731

[121] Scribner JA Hav M Summers A Nguyen H Conner J Corey E . Abstract A140: MGC026, a glycan-linked, exatecan-based antibody-drug conjugate (ADC) targeting B7-H3, is efficacious toward prostate cancer patient-derived xenografts. Mol Cancer Ther. (2025) 24(10_Supplement).

[122] Lutz MS Zekri L Weßling L Berchtold S Heitmann JS Lauer UM . IgG-based B7-H3xCD3 bispecific antibody for treatment of pancreatic, hepatic and gastric cancer. Front Immunol. (2023) 14. 10.3389/fimmu.2023.1163136 PMC10140336 37122707

[123] Powderly J Cote G Flaherty K Szmulewitz RZ Ribas A Weber J . Interim results of an ongoing Phase I, dose escalation study of MGA271 (Fc-optimized humanized anti-B7-H3 monoclonal antibody) in patients with refractory B7-H3-expressing neoplasms or neoplasms whose vasculature expresses B7-H3. J Immunother Cancer. (2015) 3(Suppl 2).

[124] Modak S Zanzonico P Grkovski M Slotkin EK Carrasquillo JA Lyashchenko SK . B7H3-directed intraperitoneal radioimmunotherapy with radioiodinated omburtamab for desmoplastic small round cell tumor and other peritoneal tumors: results of a phase I study. J Clin Oncol. (2020) 38:4283–91. 10.1200/JCO.20.01974 PMC7768336 33119478

[125] Shenderov E Mallesara GHG Wysocki PJ Xu W Ramlau R Weickhardt AJ . 620P MGC018, an anti-B7-H3 antibody-drug conjugate (ADC), in patients with advanced solid tumors: Preliminary results of phase I cohort expansion. Ann Oncol. (2021) 32:S657–9.

[126] Jang S Powderly JD Spira AI Bakkacha O Loo D Bohac GC . Phase 1 dose escalation study of MGC018, an anti-B7-H3 antibody-drug conjugate (ADC), in patients with advanced solid tumors. J Clin Oncol. (2021) 39(15_suppl).

[127] Carneiro BA Perets R Dowlati A LoRusso P Yonemori K He L . Mirzotamab clezutoclax as monotherapy and in combination with taxane therapy in relapsed/refractory solid tumors: Dose expansion results. J Clin Oncol. (2023) 41(16_suppl).

[128] Shen L Meng X Sun Y Zhao Y Huang H Han L . Promising early results of MHB088C (B7-H3 ADC) in patients (pts) with metastatic castration-resistant prostate cancer (mCRPC) from a phase 1/2 multicenter study. J Clin Oncol. (2025) 43(16_suppl).

[129] Shen L Zhou C Yu Y Yu X Meng X Sun Y . Efficacy and safety of MHB088C, a novel B7-H3-targeted ADC, in patients with relapsed extensive-stage small cell lung cancer (ES-SCLC): Subgroup analysis from a phase 1/2 multicenter study. J Clin Oncol. (2025) 43(16_suppl).

[130] Zhang Z Liang X Huang Y Yang L Jiang H Qin Y . Results from a phase 1/2 study of 7MW3711: A novel B7-H3 antibody-drug conjugate (ADC) incorporating a topoisomerase I inhibitor in patients with advanced solid tumors. J Clin Oncol. (2025) 43(16_suppl).

[131] Vitanza NA Wilson AL Huang W Seidel K Brown C Gustafson JA . Intraventricular B7-H3 CAR T cells for diffuse intrinsic pontine glioma: preliminary first-in-human bioactivity and safety. Cancer Discov. (2023) 13:114–31. 10.1158/2159-8290.CD-22-0750 PMC9827115 36259971

[132] Pinto N Albert CM Taylor MR Ullom HB Wilson AL Huang W . STRIvE-02: A first-in-human phase I study of systemically administered B7-H3 chimeric antigen receptor T cells for patients with relapsed/refractory solid tumors. J Clin Oncol. (2024) 42:4163–72. 10.1200/JCO.23.02229 39255444

